# Comparative efficacy and safety of core decompression, cell-based therapy, hyperbaric oxygen therapy, extracorporeal shock wave therapy, and combined regimens for osteonecrosis of the femoral head: a network meta-analysis

**DOI:** 10.3389/fcell.2026.1876711

**Published:** 2026-07-15

**Authors:** Qian-yu Niu, Xiao-qiang Yang, Xin-wei Liu, Fan Yang, Wei He

**Affiliations:** 1 Guangzhou University of Chinese Medicine, Guangzhou, China; 2 The Third Affiliated Hospital of Guangzhou University of Chinese Medicine, Guangzhou, China; 3 State Key Laboratory of Traditional Chinese Medicine Syndrome, Guangzhou, China

**Keywords:** bone marrow aspirate concentrate, cell therapy, core decompression, extracorporeal shock wave therapy, hyperbaric oxygen therapy, network meta-analysis, osteonecrosis of the femoral head

## Abstract

**Background:**

Osteonecrosis of the femoral head (ONFH) is a progressive, disabling disorder affecting young and middle-aged adults. Joint-preserving treatments—core decompression (CD), cell-based therapy, extracorporeal shock wave therapy (ESWT), hyperbaric oxygen therapy (HBO), and combined regimens—are increasingly used in early-to mid-stage ONFH, but their comparative efficacy and safety remain uncertain. Cellular therapies, varying in source, preparation, dose, and delivery, were analyzed as a single node to preserve network connectivity.

**Methods:**

This PRISMA-NMA–compliant network meta-analysis was prospectively registered in PROSPERO. PubMed, Embase, Web of Science, and Cochrane Library were searched through 20 April 2026. RCTs and retrospective comparative studies in adults with ONFH were eligible. Primary outcomes were visual analogue scale Harris Hip Score and imaging progression; secondary outcomes included total hip arthroplasty conversion, Oxford Hip Score SF-36, WOMAC, and adverse events. Random-effects models were used primarily, with fixed-effect models applied to sparse networks. Treatment rankings were estimated via SUCRA; risk of bias was assessed with RoB 2.0 and ROBINS-I.

**Results:**

Twenty-nine studies (2,177 hips) were included. HBO-based interventions, particularly HBO plus CD, showed the greatest pain reduction. CD plus ESWT ranked highest for HHS improvement, though substantial network inconsistency limited confidence in this ranking. For imaging progression, CD combined with cell therapy and ESWT significantly reduced radiographic deterioration, with CD plus cell therapy ranking highest. Heterogeneity in cell source, preparation, dose, and delivery precludes identification of an effective product or protocol. No intervention significantly reduced THA conversion. HBO plus CD ranked highest for OHS and SF-36, but these networks were sparse and estimates preliminary. ESWT showed the most favorable safety profile, although the safety endpoint combined events of markedly differing clinical severity. WOMAC results were highly heterogeneous and unstable in sensitivity analyses.

**Conclusion:**

Joint-preserving treatments for ONFH showed outcome-specific advantages rather than a uniform hierarchy. HBO-based therapy appeared most favorable for pain and quality of life, CD plus ESWT for function, and CD plus cell therapy or ESWT for structural preservation. The efficacy of specific cell products or protocols remains uncertain. High-quality randomized trials with standardized protocols, harmonized outcomes, and long-term follow-up are needed.

**Systematic Review Registration:**

https://www.crd.york.ac.uk/PROSPERO/view/CRD420261379078, identifier PROSPERO CRD420261379078.

## 1 Introduction

Osteonecrosis of the femoral head (ONFH) is a progressive disorder caused by impaired blood supply to the femoral head, leading to osteocyte death, subchondral fracture, articular surface collapse, and ultimately degenerative hip failure (Zhao et al., 2020; Cohen-Rosenblum and Cui, 2019; Hines et al., 2021). The disease commonly affects young and middle-aged adults. Pain, impaired mobility, reduced work capacity, and loss of quality of life may develop long before the age at which primary total hip arthroplasty (THA) is typically indicated (Zhao et al., 2020; Cohen-Rosenblum and Cui, 2019).

The social and economic burden of ONFH is therefore substantial. Patients often require prolonged conservative care, repeated imaging surveillance, joint-preserving procedures, or eventual THA; for younger patients, arthroplasty also raises concerns regarding implant longevity, revision burden, and cumulative lifetime surgical risk (Zhao et al., 2020; Buddhiraju et al., 2025; Qi et al., 2025). These considerations have made early diagnosis, accurate staging, and effective joint-preserving treatment central goals in contemporary ONFH management (Hines et al., 2021; Yoon et al., 2020; Steinberg et al., 1995; Pierce et al., 2015; Sultan et al., 2019).

Several joint-preserving interventions are currently used in early- and mid-stage ONFH. Core decompression (CD) aims to reduce intraosseous pressure, improve local perfusion, and facilitate reparative tissue ingrowth (Hua et al., 2019). Cell therapy has been introduced to enhance local osteogenesis and angiogenesis; published studies have employed biologically distinct products, including bone marrow aspirate concentrate (BMAC), bone marrow mesenchymal stem cells, bone marrow mononuclear cells, and autologous bone marrow–derived osteoblastic cells (Andronic et al., 2021; Pawar et al., 2022; Jindal et al., 2021; Hoogervorst et al., 2022; Saini et al., 2023; Migliorini et al., 2021). In this network meta-analysis, these heterogeneous strategies were collectively analyzed under the label BMAC—a grouping adopted to maintain network connectivity rather than to imply biological equivalence. Hyperbaric oxygen therapy (HBO) has been proposed to improve tissue oxygenation and microcirculation (Uzun et al., 2016; Ansari et al., 2025), and extracorporeal shock wave therapy (ESWT) may promote neovascularization, modulate pain signaling, and stimulate bone remodeling (Hao et al., 2018; Sconza et al., 2022; Luan et al., 2022; Yang et al., 2022).

Previous clinical studies and conventional meta-analyses have suggested potential benefits of these interventions, but the evidence remains fragmented. Most studies compare a single active treatment with CD, placebo, or conservative care; direct head-to-head evidence among CD, cell therapy, HBO, ESWT, and combined regimens is limited (Tang et al., 2025; Hu et al., 2023; Li et al., 2023). Pairwise meta-analysis therefore cannot provide a complete comparative hierarchy across the multiple competing joint-preserving strategies evaluated in partially connected evidence networks.

Network meta-analysis (NMA) offers a methodological framework to synthesize both direct and indirect evidence and to estimate comparative treatment rankings within a connected network (Chaimani et al., 2013; White, , 2015). This approach is particularly suited to ONFH, where multiple joint-preserving strategies and combination regimens have been evaluated but direct comparative trials remain scarce.

Accordingly, this systematic review and NMA compared the efficacy and safety of CD, cell therapy (coded as BMAC), HBO, ESWT, and their combined regimens in patients with ONFH. The primary outcomes were VAS, HHS, and imaging progression; secondary outcomes were THA conversion, OHS, SF-36, WOMAC, and treatment-related adverse events. By integrating randomized and retrospective comparative evidence, this study aimed to clarify the relative clinical, radiological, and safety profiles of available joint-preserving strategies and to identify areas in which higher-quality comparative evidence is still needed.

## 2 Methods

### 2.1 Registration and reporting standards

This NMA was conducted in accordance with the PRISMA extension statement for Network Meta-Analyses (PRISMA-NMA) (Cameron et al., 2015). The protocol was prospectively registered in PROSPERO (CRD420261379078) (Hutton et al., 2015).

### 2.2 Literature search strategy and outcome definition

A systematic search of PubMed, Embase, Web of Science, and the Cochrane Library was performed for English-language studies from database inception to 20 April 2026. The search strategy combined controlled vocabulary and free-text terms including “osteonecrosis,” “femoral head,” “core decompression,” “extracorporeal shock wave therapy,” “hyperbaric oxygen therapy,” and “cell therapy.” The full search strategy is provided in Supplementary Material 1. Imaging progression was defined as radiographic evidence of femoral head collapse, advancement of disease stage (by Ficat, ARCO, or Steinberg classification), progressive loss of femoral head sphericity on serial imaging, or any other definition of structural deterioration reported by the original study. The specific definition used in each included study was extracted during data collection; however, formal cross-study harmonization was limited by between-study heterogeneity in imaging modalities, follow-up intervals, and reporting standards.

### 2.3 Eligibility criteria

Studies were eligible if they met the following criteria (Zhao et al., 2020): randomized controlled trials (RCTs) or retrospective comparative cohort studies (Cohen-Rosenblum and Cui, 2019); adult patients (≥18 years) with ONFH, predominantly Steinberg I–II, ARCO or Ficat stages I–III, with a small proportion of ARCO stage IV patients (Yoon et al., 2020; Steinberg et al., 1995; Sultan et al., 2019); (Hines et al., 2021) evaluation of CD, CD plus cell therapy, ESWT, HBO, placebo or conservative control, or combined strategies (CD plus ESWT, HBO plus CD, ESWT plus HBO); and (Buddhiraju et al., 2025) reporting of at least one eligible clinical, radiological, quality-of-life, or safety outcome. Because cellular interventions differed in cell source, preparation, expansion, and delivery protocol, all cell-based strategies—including BMAC, bone marrow mesenchymal stem cells, bone marrow mononuclear cells, and autologous bone marrow-derived osteoblastic cells—were coded under the unified analytical label BMAC (Xu et al., 2024; Ulusoy et al., 2023; Jayankura et al., 2023; Tomaru et al., 2021; Martinot et al., 2020; Li et al., 2020; Nally et al., 2018; Kang et al., 2018; Hernigou et al., 2018; Hauzeur et al., 2018; Pilge et al., 2016; Gianakos et al., 2016; Yan et al., 2015; Tabatabaee et al., 2015; Ma et al., 2014; Sen et al., 2012; Zhao et al., 2012; Gangji et al., 2011). This pragmatic grouping was required to preserve a connected treatment network; in preliminary analyses, separating these interventions into distinct nodes resulted in network fragmentation. The BMAC label therefore represents a statistical necessity rather than a claim of biological equivalence. Intervention details are provided in Supplementary Material 2. Exclusion criteria were: animal or *in vitro* studies; case reports; protocols; reviews; letters; editorials; conference abstracts; duplicate publications; studies with unavailable full text, missing or seriously erroneous data, or insufficient follow-up; and studies not published in English.

### 2.4 Data extraction

All retrieved records were managed using EndNote 21. Two reviewers (QN and XY) independently screened titles and abstracts, followed by full-text assessment according to the predefined eligibility criteria. Disagreements were resolved by discussion or consultation with a third reviewer (WH). Two reviewers independently extracted study information using a standardized spreadsheet, including first author, publication year, country or region, study design, intervention and comparator, baseline participant characteristics, sample size, follow-up duration, and eligible outcome data. Nonpharmacological interventions and control groups were independently categorized by two reviewers, with discrepancies resolved by consensus. When reported, protocol-level information was also extracted and summarized in Supplementary Material 2 (including HBO chamber pressure, session duration and number; ESWT energy flux density, shock number, session number, and guidance method; CD drilling technique and graft or adjunct use; and cell source, processing method, dose, expansion status, and delivery route).

### 2.5 Risk of bias assessment and GRADE certainty of evidence

Risk of bias was assessed according to study design. RCTs were evaluated using the Cochrane Risk of Bias two tool (RoB 2.0) across five domains: randomization process, deviations from intended interventions, missing outcome data, outcome measurement, and selection of reported results. Retrospective studies were assessed using ROBINS-I across seven domains: confounding, selection of participants, classification of interventions, deviations from intended interventions, missing data, outcome measurement, and selection of reported results. Two reviewers independently assessed each study; disagreements were resolved by discussion or adjudication by a third reviewer (Sterne et al., 2019). For RCTs, blinding of participants and personnel was considered infeasible for most comparisons because of the substantial physical and procedural differences among surgical, injection-based, and noninvasive interventions. Lack of blinding was therefore treated as a potential source of performance bias, particularly for subjective outcomes (VAS, HHS, OHS, SF-36, WOMAC). Allocation concealment was evaluated separately from blinding; studies with unclear or absent allocation concealment were flagged. For retrospective studies, confounding by indication, baseline disease severity, lesion characteristics, and center-level practice patterns were considered key sources of bias. These concerns were incorporated into the GRADE assessment through downgrading for risk of bias, inconsistency, indirectness, or imprecision where applicable. The certainty of evidence for each outcome was evaluated according to the GRADE framework (Balshem et al., 2011), considering risk of bias, inconsistency, indirectness, imprecision, and other considerations (see Table 1).

**TABLE 1:** GRADE assessment of the certainty of evidence for each outcome.

Outcome	Studies, n	Study design	Risk of bias	Inconsistency	Indirectness	Imprecision	Other	Certainty
VAS	17	RCT/cohort	Serious	Serious	Not serious	Serious	Publication bias suspected	Low
HHS	13	RCT/cohort	Serious	Serious	Serious	Not serious	Publication bias suspected	Low
Imaging progression	12	RCT/cohort	Not serious	Not serious	Serious	Serious	Undetected	Low
THA conversion	17	RCT/cohort	Serious	Not serious	Serious	Serious	Publication bias suspected	Low
OHS	3	RCT	Serious	Not serious	Not serious	Serious	None	Low
SF-36	3	RCT	Serious	Serious	Not serious	Serious	Undetected	Very low
Adverse events	10	RCT/cohort	Serious	Not serious	Serious	Serious	Publication bias suspected	Low
WOMAC	4	RCT/cohort	Serious	Very serious	Serious	Serious	Publication bias strongly suspected	Very low

### 2.6 Statistical analysis

Network meta-analysis was performed using Stata version 18.0 (Chaimani et al., 2013; White, , 2015). Odds ratios (ORs) were used for dichotomous outcomes; mean differences (MDs) or standardized mean differences (SMDs) were used for continuous outcomes, with 95% confidence intervals (CIs). Random-effects consistency models were used as the primary analytical approach for all outcomes. For outcomes supported by sparse networks (three or fewer studies), fixed-effect inconsistency models were applied because between-study heterogeneity cannot be reliably estimated from very limited evidence; this applied to the OHS and SF-36 networks. For WOMAC, where only two interventions were available, a conventional pairwise random-effects meta-analysis was performed using the DerSimonian–Laird method (DerSimonian and Laird, 1986).

Consistency between direct and indirect evidence was assessed using global inconsistency tests, node-splitting analysis, and loop-specific inconsistency factors where applicable (Dias et al., 2010). Treatment rankings were estimated using the surface under the cumulative ranking curve (SUCRA); values closer to 100% indicate a greater probability of being the most favorable intervention (Salanti et al., 2011). League tables were generated for all pairwise comparisons. Potential small-study effects and publication bias were assessed by visual inspection of comparison-adjusted funnel plots when the number of contributing studies was sufficient (Chaimani et al., 2013); for sparse networks, funnel plot findings were assessed with recognition of their limited power to detect or exclude small-study effects.

To evaluate the robustness of the primary findings, three additional sensitivity analyses were conducted: (a) an analysis restricted to RCTs; (b) an analysis excluding studies with follow-up shorter than 24 months for the structural outcomes of imaging progression and THA conversion; and (c) a subgroup analysis limited to studies enrolling patients with early-stage disease (Ficat I–II or ARCO I–II). All sensitivity analyses used the same random-effects consistency model as the primary analysis. Quantitative meta-regression adjusting for disease stage, follow-up duration, lesion size, etiology, or treatment protocol was planned but was not feasible owing to insufficient covariate variation and incomplete reporting across studies.

## 3 Results

### 3.1 Study selection

**FIGURE 1:**
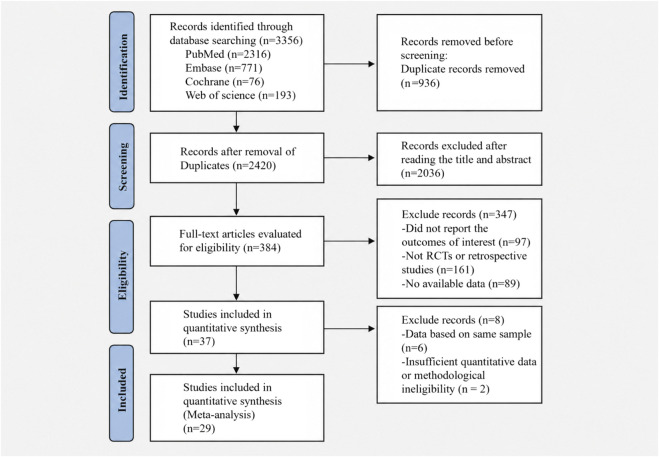
PRISMA flow diagram of study selection.

The literature search identified 3,356 records. After removal of 936 duplicates, 2,420 records underwent title and abstract screening. Of these, 2,036 were excluded. Full texts were assessed for 384 articles; 347 were excluded because they did not report outcomes of interest, were not RCTs or retrospective comparative studies, or did not provide usable data. Among 37 studies initially eligible for quantitative synthesis, eight were further excluded because of overlapping samples, insufficient quantitative data, or failure to meet the minimum methodological standards for inclusion in a contemporary evidence synthesis. Ultimately, 29 studies were included in the NMA (see Figure 1).

### 3.2 Study characteristics

The 29 included studies were published between 2010 and 2026 and comprised 17 RCTs (Xu et al., 2024; Ulusoy et al., 2023; Jayankura et al., 2023; Tomaru et al., 2021; Martinot et al., 2020; Li et al., 2020; Nally et al., 2018; Kang et al., 2018; Hernigou et al., 2018; Sun et al., 2026; Zhu et al., 2023; Liu et al., 2023; Yang et al., 2022; Wang et al., 2022; Shi et al., 2022; Bozkurt et al., 2022; Moghamis et al., 2021) and 12 retrospective comparative studies (Hauzeur et al., 2018; Pilge et al., 2016; Gianakos et al., 2016; Yan et al., 2015; Tabatabaee et al., 2015; Ma et al., 2014; Sen et al., 2012; Zhao et al., 2012; Gangji et al., 2011; Wang et al., 2015; Hsu et al., 2010; Camporesi et al., 2010), involving 2,177 hips. Studies were conducted across multiple countries; the largest proportion originated from China, followed by Turkey, France, Belgium, and the United States. The evaluated interventions included CD, CD plus cell therapy (CD plus BMAC), ESWT, HBO, placebo or conservative control, and combination regimens (HBO plus CD, CD plus ESWT, ESWT plus HBO). Assessed outcomes included VAS, HHS, imaging progression, THA conversion, OHS, SF-36, WOMAC, and treatment-related adverse events (see Table 2).

**TABLE 2:** Baseline characteristics of the included studies.

First author (year)	Country	Intervention	Sample (n)	Male/Female	Age, y	BMI	Stage	Follow-up Mon	Outcomes
Sun et al. (2026)	China	HBO + CD	20	16/4	44.10 ± 4.33	24.4 ± 3.59	Ficat II	19.65 ± 2.13	1,2,4
​	​	CD	20	15/5	44.30 ± 4.58	NA	Ficat II	20.05 ± 2.24	1,2,4
​	​	HBO	20	14/6	44.30 ± 4.58	NA	Ficat II	19.10 ± 2.15	1,2,4
Xu et al. (2024)	China	CD + BMAC	29	18/11	44.66 ± 10.98	NA	Ficat I-II	NA	3,6,7
​	​	P	20	13/7	42.15 ± 11.59	NA	Ficat I-II	NA	3,6,7
Zhu et al. (2023)	China	ESWT	35	18/17	43.72 ± 13.69	NA	ARCO I-IV	2	6,7,5
​	​	P	35	25/10	40.06 ± 13.48	NA	ARCO I-IV	2	6,7,5
Ulusoy et al. (2023)	Turkey	CD + BMAC	19	NA	38.4 ± 6.7	NA	Steinberg I-II	32.2 ± 4.1	6,7
​	​	CD	25	NA	39.3 ± 6.5	NA	Steinberg I-II	31.85 ± 4.4	6,7
Liu et al. (2023)	China	CD + ESWT	53	41/12	45.85 ± 6.01	23.9 ± 2.7	ARCO I-II	31.06 ± 4.28	6,7
​	​	ESWT	53	34/19	45.12 ± 5.83	23.5 ± 2.5	ARCO I-II	31.06 ± 4.28	6,7
Jayankura et al. (2023)	Europe	CD + BMAC	25	NA	46 ± 10	NA	ARCO I-II	24	3,8
​	​	CD	29	NA	45 ± 10	NA	ARCO I-II	24	3,8
Yang et al. (2022)	China	ESWT	75	29/46	45.83 ± 13.10	NA	ARCO I-II	12	4
​	​	P	78	35/43	44.18 ± 12.85	NA	ARCO I-II	12	4
Wang et al. (2022)	China	CD	67	55/16	38.6 ± 9.8	24.4 ± 3.59	ARCO I-III	60	1,3,4
​	​	P	50	41/12	39.0 ± 9.7	NA	ARCO I-III	60	1,3,4
Shi et al. (2022)	China	ESWT	73	43/40	47.9 ± 15.3	NA	ARCO I-III	12	6,7
​	​	P	70	38/32	46.0 ± 14.7	NA	ARCO I-III	12	6,7
Bozkurt et al. (2022)	Turkey	HBO + CD	34	24/10	39.2 ± 8.7	NA	Ficat II	43.1	2,6,7
​	​	HBO	46	30/16	39.9 ± 9.6	NA	Ficat II	39.8	2,6,7
Tomaru et al. (2021)	Japan	CD + BMAC	33	2/31	35.1	NA	ARCO I-IIIB	5.9	3
​	​	P	33	2/31	35.7	NA	ARCO I-IIIB	8.7	3
Moghamis et al. (2021)	Qatar	CD	12	5/5	35.4 ± 10.5	29.1 ± 3.3	Steinberg II	48.7 ± 13.3	1,2,4
​	​	HBO	11	4/5	35.1 ± 9.5	25.2 ± 4.5	Steinberg II	18.2 ± 4.2	1,2,4
Martinot et al. (2020)	France	CD	24	19/5	41.06 ± 9.53	26.27 ± 3.78	Ficat I-III	64 ± 64.5	3,7
​	​	CD + BMAC	43	36/7	43.23 ± 10.97	25.31 ± 4.17	Ficat I-III	64 ± 64.5	3,7
Li et al. (2020)	China	CD	14	10/4	NA	NA	Ficat II-III	60	3,5,6
​	​	CD + BMAC	17	12/5	NA	NA	Ficat II-III	60	3,5,6
Nally et al. (2018)	Italy	CD	33	9/24	38	NA	Ficat I-II	72	3
​	​	CD + BMAC	39	10/29	41	NA	Ficat I-II	72	3
Kang et al. (2018)	Korea	CD	50	38/12	47.3 ± 9.7	24.0 ± 4.1	ARCO I-IV	48	3,4
​	​	CD + BMAC	50	36/14	46.0 ± 9.3	23.8 ± 3.7	ARCO I-IV	54	3,4
Hernigou et al. (2018)	France	CD	125	NA	NA	NA	Steinberg I-II	300	3,4,6,7
​	​	CD + BMAC	125	NA	NA	NA	Steinberg I-II	300	3,4,6,7
Hauzeur et al. (2018)	Belgium	CD	19	13/6	49.7 ± 3.2	24.53 ± 0.96	ARCO III	24	3,5,6
​	​	CD + BMAC	19	14/5	48.0 ± 2.8	25.35 ± 0.75	ARCO III	24	3,5,6
Pilge et al. (2016)	Germany	CD + BMAC	10	9/1	NA	NA	ARCO II-IV	34.1	3
​	​	CD	10	8/2	NA	NA	ARCO II-IV	27	3
Gianakos et al. (2016)	United States	CD + BMAC	20	11/9	38 ± 14.7	NA	Ficat I-II	25.3 ± 11.5	3,6,7
​	​	P	29	11/18	43 ± 12.1	NA	Ficat I-II	22.7 ± 19.5	3,6,7
Yan et al. (2015)	China	CD	42	20/22	37.24 ± 10.54	NA	ARCO I-II	26.45 ± 1.26	4,6,7
​	​	CD + BMAC	44	23/21	39.62 ± 11.83	NA	ARCO I-II	26.24 ± 1.32	4,6,7
Wang et al. (2015)	China	ESWT	23	20/3	39.8 ± 12.1	NA	ARCO I-III	25.2 ± 3.7	3,6,7
​	​	CD	25	23/2	39.9 ± 9.3	NA	ARCO I-III	25.8 ± 4.6	3,6,7
Tabatabaee et al. (2015)	Iran	CD + BMAC	14	9/5	31 ± 11.4	NA	ARCO I-III	24	3,5,6
​	​	CD	14	10/4	26.8 ± 5.8	NA	ARCO I-III	24	3,5,6
Ma et al. (2014)	China	CD	18	13/5	34.78 ± 11.48	NA	Ficat I-III	24	6,5
​	​	CD + BMAC	21	15/6	35.60 ± 8.05	NA	Ficat I-III	24	6,5
Shan-Ling Hsu et al. (2010)	China	ESWT + HBO	28	18/10	39.1 ± 12.6	NA	ARCO I-III	25.6 ± 11.0	3,4,5,6,7
​	​	ESWT	35	27/8	39.6 ± 11.9	NA	ARCO I-III	29.0 ± 12.4	3,4,5,6,7
Enrico Camporesi et al. (2010)	United States of America	HBO	10	6/4	49	NA	Ficat II	84	6
​	​	P	9	6/3	48.8	NA	Ficat II	84	6
Ramesh Kumar Sen et al. (2012)	India	CD	25	18/7	NA	NA	ARCO I-II	24	7
​	​	CD + BMAC	26	19/7	NA	NA	ARCO I-II	24	7
Zhao et al. (2012)	China	CD	43	19/24	33.8 ± 7.70	NA	ARCO I-II	60	3,4,7
​	​	CD + BMAC	50	27/23	32.7 ± 10.5	NA	ARCO I-II	60	3,4,7
Gangji et al. (2011)	Belgium	CD	9	NA	45.7 ± 2.8	NA	ARCO I-II	60	3,4,6,8
​	​	CD + BMAC	10	NA	42.2 ± 2.6	NA	ARCO I-II	60	3,4,6,8

Outcome codes: 1 = Oxford Hip Score (OHS); 2 = SF-36; 3 = rate of THA; 4 = imaging progression evaluation; 5 = Western Ontario and McMaster Universities Osteoarthritis Index (WOMAC); 6 = visual analogue scale (VAS); 7 = Harris Hip Score (HHS); 8 = adverse events. Stage refers to the classification reported in the original study; P indicates placebo or conservative control; BMAC, denotes the pooled analytical category for cell therapy.

### 3.3 Risk of bias assessment

**FIGURE 2:**
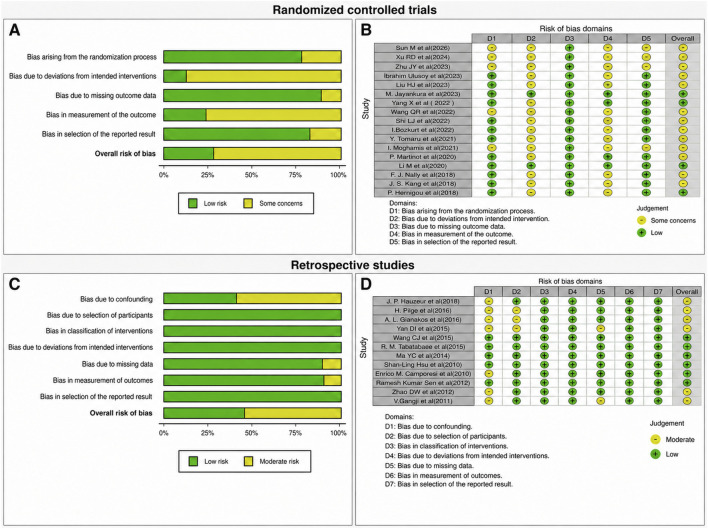
Risk-of-bias assessment for randomized controlled and retrospective studies. **(A)** Summary plot of risk of bias across domains for randomized controlled trials. **(B)** Traffic-light plot showing domain-level risk-of-bias judgments for individual randomized controlled trials. **(C)** Summary plot of risk of bias across domains for retrospective studies. **(D)** Traffic-light plot showing domain-level risk-of-bias judgments for individual retrospective studies.

Most RCTs showed low risk of bias in domains related to randomization, missing outcome data, and selective reporting. However, as shown in Figure 2, many RCTs were judged as having some concerns for deviations from intended interventions or outcome measurement because blinding of participants, surgeons, or treating personnel to physically distinct interventions was usually infeasible. Performance bias therefore could not be excluded for subjective outcomes. Retrospective studies showed more variability; several were judged as having moderate risk of bias, mainly owing to potential confounding by indication, baseline disease severity, lesion characteristics, and participant selection. Intervention classification and outcome measurement were generally acceptable across the retrospective evidence base. Overall, RCTs contributed relatively higher-quality evidence, whereas retrospective studies increased the breadth of available comparisons but introduced non-randomized-study limitations that were incorporated into the GRADE assessment (see Figure 2; Table 1).

### 3.4 Results of the network meta-analysis

#### 3.4.1 Network geometry

**FIGURE 3:**
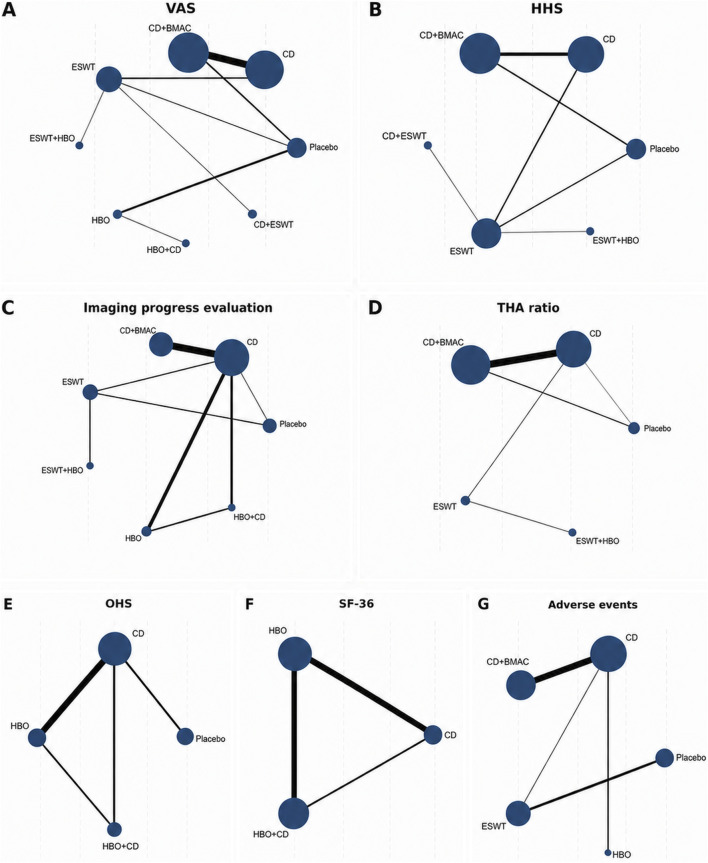
Network geometry of the analyzed treatment comparisons. **(A)** VAS. **(B)** HHS. **(C)** Imaging progression evaluation. **(D)** THA conversion ratio. **(E)** OHS. **(F)** SF-36. **(G)** Adverse events. Nodes represent interventions, and edges represent direct comparisons; thicker edges indicate a larger number of direct comparisons.

Separate evidence networks were constructed for VAS, HHS, imaging progression, THA conversion, OHS, SF-36, and adverse events. Nodes represented interventions (node size reflecting the amount of evidence) and edges represented direct head-to-head comparisons (thicker edges indicating more direct comparisons). The networks were connected for all assessed outcomes, supporting the feasibility of NMA. Across outcomes, CD, CD plus BMAC, and ESWT served as key connecting interventions, particularly within the VAS, HHS, imaging progression, and THA conversion networks (see Figure 3).

##### 3.4.1.1 VAS

**FIGURE 4:**
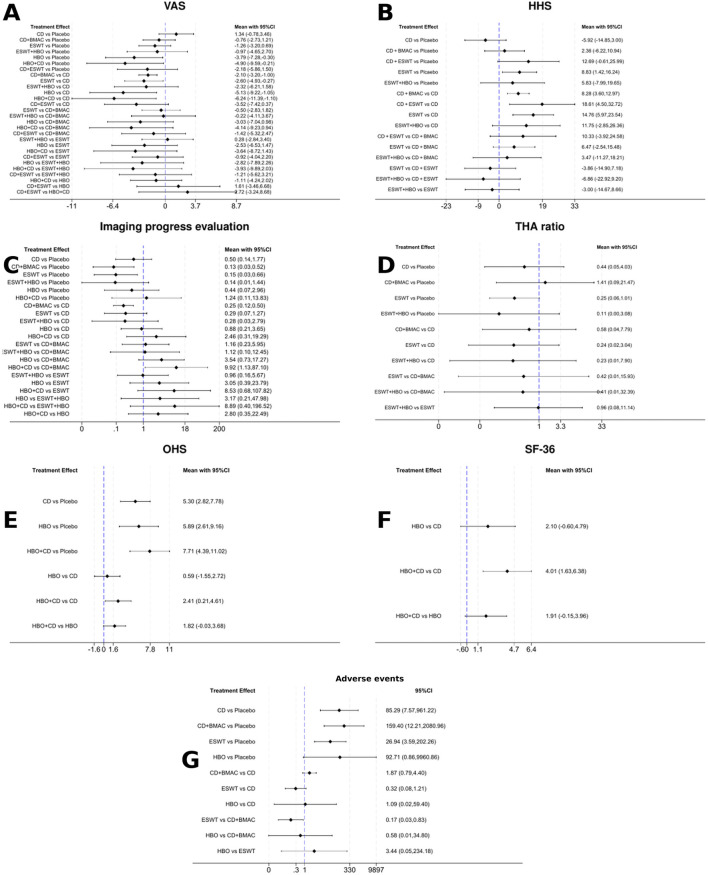
Forest plots of comparative effect estimates. **(A)** VAS. **(B)** HHS. **(C)** Imaging progression evaluation. **(D)** THA conversion ratio. **(E)** OHS. **(F)** SF-36. **(G)** Adverse events. Points represent effect estimates, and horizontal lines represent 95% confidence intervals.

**FIGURE 5:**
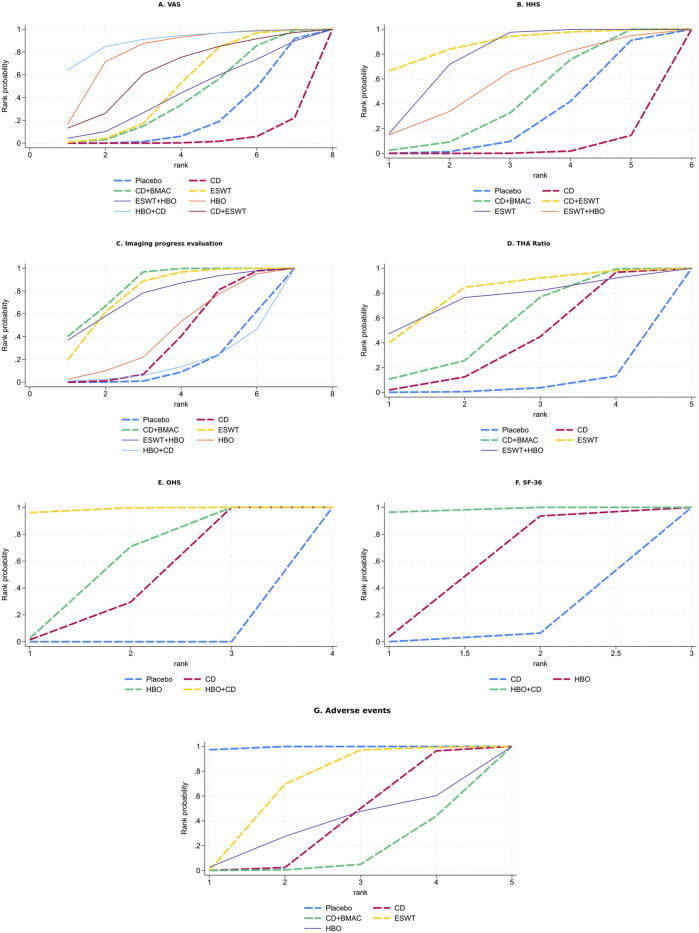
SUCRA ranking curves for the analyzed outcomes. **(A)** VAS. **(B)** HHS. **(C)** Imaging progression evaluation. **(D)** THA conversion ratio. **(E)** OHS. **(F)** SF-36. **(G)** Adverse events.

In the VAS network, lower values indicated greater pain relief. Compared with placebo, HBO and HBO plus CD showed significant reductions in VAS (SMD: −3.79, 95% CI: −7.28 to −0.30; and SMD: −4.90, 95% CI: −9.59 to −0.21, respectively). Compared with CD alone, CD plus BMAC, ESWT, HBO, and HBO plus CD all showed significant additional reductions (SMD: −2.10, 95% CI: −3.20 to −1.00; SMD: −2.60, 95% CI: −4.93 to −0.27; SMD: −5.13, 95% CI: −9.22 to −1.05; and SMD: −6.24, 95% CI: −11.39 to −1.10, respectively) (see Figure 4A; Supplementary Table S1). SUCRA ranking indicated that HBO plus CD had the highest probability of being the most favorable treatment for VAS improvement, followed by HBO and CD plus ESWT (see Figure 5A; Supplementary Table S8).

##### 3.4.1.2 HHS

In the HHS network, higher values indicated better hip function. Compared with placebo, ESWT showed a significant improvement (MD: 8.83, 95% CI: 1.42–16.24). Compared with CD alone, CD plus BMAC, CD plus ESWT, and ESWT showed significant functional advantages (MD: 8.28, 95% CI: 3.60 to 12.97; MD: 18.61, 95% CI: 4.50 to 32.72; and MD: 14.76, 95% CI: 5.97 to 23.54, respectively) (see Figure 4B; Supplementary Table S2). SUCRA ranking favored CD plus ESWT, followed by ESWT and ESWT plus HBO (see Figure 5B; Supplementary Table S9). Importantly, substantial network inconsistency was detected for HHS (loop inconsistency factor, 16.82; 95% CI: 2.26–31.37); the HHS rankings therefore carry substantial uncertainty; the underlying effect estimates and GRADE certainty ratings provide a more reliable basis for inference.

##### 3.4.1.3 Imaging progression

For imaging progression, lower estimates indicated lower risk of radiographic deterioration. Compared with placebo, CD plus BMAC and ESWT showed significantly lower risks (OR: 0.13, 95% CI: 0.03 to 0.52; and OR: 0.15, 95% CI: 0.03 to 0.66, respectively). Compared with CD alone, CD plus BMAC also showed a significant reduction (OR: 0.25, 95% CI: 0.12–0.50). Among active interventions, HBO plus CD showed a higher risk than CD plus BMAC (OR: 9.92, 95% CI: 1.13–87.10) (see Figure 4C; Supplementary Table S3). SUCRA ranking placed CD plus BMAC first, followed by ESWT and ESWT plus HBO (see Figure 5C; Supplementary Table S10). Because BMAC is an umbrella analytical category encompassing heterogeneous cell products, this finding does not identify which specific cell product, preparation method, or dose produced the observed benefit. The pooled BMAC effect estimate reflects a weighted average across biologically distinct cell-based interventions—including mesenchymal stem cells, mononuclear cells, cultured stem cells, and osteoblastic-cell preparations—and should not be interpreted as evidence supporting any specific cell type, preparation method, or dose.

##### 3.4.1.4 Conversion to THA

No statistically significant differences in THA conversion were detected among the evaluated interventions, either versus placebo or among active strategies (see Figure 4D; Supplementary Table S4). Although SUCRA ranking suggested that ESWT, ESWT plus HBO, and CD plus BMAC had relatively favorable probabilities, given the non-significant network estimates and the strong dependence of THA conversion on disease stage, surgeon decision-making, patient factors, and follow-up duration, the SUCRA probabilities alone are insufficient to establish clinical superiority (see Figure 5D; Supplementary Table S11).

##### 3.4.1.5 OHS

Compared with placebo, CD, HBO, and HBO plus CD showed significant improvements in OHS (MD: 5.30, 95% CI: 2.82 to 7.78; MD: 5.89, 95% CI: 2.61 to 9.16; and MD: 7.71, 95% CI: 4.39 to 11.02, respectively). Compared with CD alone, HBO plus CD showed a significant additional improvement (MD: 2.41, 95% CI: 0.21–4.61) (see Figure 4E; Supplementary Table S5). SUCRA ranking placed HBO plus CD first, followed by HBO and CD; however, only three studies contributed to the OHS network; rankings for HBO-based strategies should therefore be regarded as preliminary and interpreted with caution, given the limited evidence base of only three studies and the potential instability of network estimates derived from sparse data (see Figure 5E; Supplementary Table S12).

##### 3.4.1.6 SF-36

Compared with CD alone, HBO plus CD showed a significant improvement in SF-36 (MD: 4.01, 95% CI: 1.63–6.38). HBO alone did not differ significantly from CD, and HBO plus CD did not differ significantly from HBO (see Figure 4F; Supplementary Table S6). SUCRA ranking placed HBO plus CD first, followed by HBO and CD; however, the SF-36 network was also limited to three studies; rankings for HBO-based strategies should therefore be regarded as preliminary and interpreted with caution, given the limited evidence base of only three studies and the potential instability of network estimates derived from sparse data (see Figure 5F; Supplementary Table S13).

##### 3.4.1.7 Adverse events

Compared with placebo, CD, CD plus BMAC, and ESWT showed significantly higher odds of adverse events (OR: 85.29, 95% CI: 7.57 to 961.22; OR: 159.40, 95% CI: 12.21 to 2080.96; and OR: 26.94, 95% CI: 3.59 to 202.26, respectively). Among active interventions, ESWT showed significantly fewer adverse events than CD plus BMAC (OR: 0.17, 95% CI: 0.03–0.83) (see Figure 4G; Supplementary Table S7). Placebo had the most favorable safety profile; ESWT ranked highest among active interventions (see Figure 5G; Supplementary Table S14). The reported adverse events encompassed heterogeneous complications of differing clinical severity (surgical complications, donor-site morbidity, post-procedural pain, ESWT-related local discomfort and petechiae, HBO-associated barotrauma and oxygen toxicity). Formal stratification by type and severity was not performed because reporting was inconsistent across studies. Consequently, the adverse-event rankings should be interpreted with considerable caution: complications of markedly different clinical severity—ranging from surgical complications and donor-site morbidity to local discomfort, petechiae, barotrauma, and oxygen toxicity—are weighted equally within a single composite endpoint. The SUCRA-based safety hierarchy should not be misinterpreted as reflecting uniform clinical severity across the reported events.

##### 3.4.1.8 WOMAC

For WOMAC, only two interventions were available; pairwise meta-analysis was performed using a random-effects DerSimonian–Laird model (DerSimonian and Laird, 1986). Four studies were included. The pooled estimate showed a significant difference between treatment and control groups (MD: 12.60, 95% CI: 2.57–22.63), with substantial between-study heterogeneity (I^2^: 99.16%; τ^2^: 102.59). Leave-one-out sensitivity analysis showed that statistical significance was not retained after excluding Li M et al. or Ma YC et al. Given the extreme heterogeneity, the presence of small-study effects, and sensitivity to individual study exclusion, these findings are provisional and require replication in larger, more homogeneous samples.

#### 3.4.2 Assessment of inconsistency, heterogeneity, and small-study effects

For VAS, the random-effects NMA showed moderate-to-substantial between-study heterogeneity (between-study SD, 1.58). Node-splitting analysis did not identify important disagreement between direct and indirect evidence. One closed loop (CD–CD plus BMAC–ESWT–placebo) was identified, with an inconsistency factor of 2.31 (95% CI: 0.00–7.04). The comparison-adjusted funnel plot showed asymmetry, suggesting potential small-study effects or publication bias.

For HHS, evidence of network inconsistency was observed. Node-splitting analysis identified disagreement between direct and indirect evidence for several comparisons (placebo vs. CD plus BMAC, placebo vs. ESWT, CD vs. CD plus BMAC, CD vs. ESWT). The closed loop involving CD, CD plus BMAC, ESWT, and placebo showed a large inconsistency factor of 16.82 (95% CI: 2.26–31.37), indicating substantial loop inconsistency. This may reflect differences in study populations, HHS measurement timing, disease stage distributions, or treatment protocols. The comparison-adjusted funnel plot showed asymmetry.

For imaging progression, no major disagreement was identified between direct and indirect evidence. Two closed loops were identified (CD–HBO–HBO plus CD; CD–placebo–ESWT). The estimated ratios of ORs were imprecise; loop-specific heterogeneity variance was <0.001 for both loops. No definite evidence of publication bias was identified, although several comparisons were supported by few studies.

For THA conversion, no important incoherence was detected. Node-splitting analysis showed no apparent disagreement between direct and indirect evidence. The comparison-adjusted funnel plot showed mild asymmetry, suggesting possible small-study effects.

For OHS, the network was sparse; a fixed-effect inconsistency model was applied. Node-splitting analysis did not identify important disagreement. The closed loop (CD–HBO–HBO plus CD) had an inconsistency factor of 1.10 (95% CI: 0.00–6.79), with no detectable loop-level heterogeneity. The comparison-adjusted funnel plot did not show marked asymmetry.

For SF-36, the network was sparse and was analyzed using a fixed-effect inconsistency model. Node-splitting analysis identified disagreement for the CD versus HBO comparison. The only closed loop (CD–HBO–HBO plus CD) had an inconsistency factor of 0.52 (95% CI: 0.00–9.27). The limited number of studies restricted formal assessment of small-study effects.

For adverse events, no closed loop was formed; loop-specific inconsistency assessment and node-splitting comparisons were therefore not applicable. The available evidence was derived mainly from a limited number of direct comparisons with wide intervals. The comparison-adjusted funnel plot showed mild dispersion and asymmetry.

For WOMAC, the funnel plot showed asymmetry, with one study far from the pooled estimate and outside the expected pseudo 95% confidence region. The Galbraith plot did not identify a clearly extreme outlier. Given the substantial heterogeneity, the observed asymmetry may reflect small-study effects, clinical heterogeneity, or methodological differences.

**FIGURE 6:**
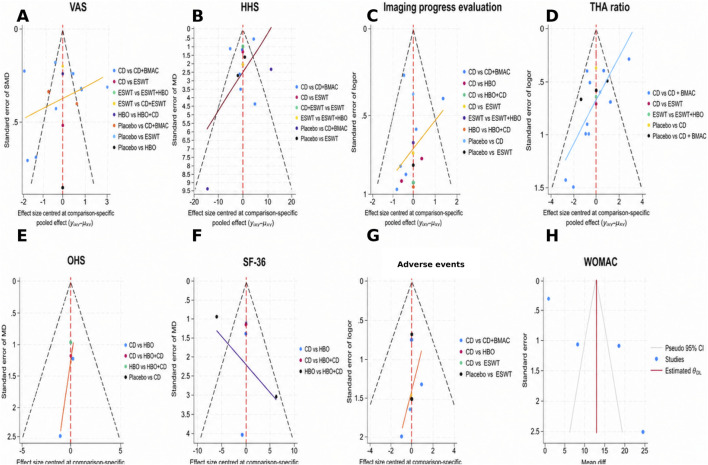
Funnel plots and small-study-effect assessments. **(A)** VAS. **(B)** HHS. **(C)** Imaging progression evaluation. **(D)** THA conversion ratio. **(E)** OHS. **(F)** SF-36. (G) Adverse events. **(H)** WOMAC.

Overall, comparison-adjusted funnel plots showed varying degrees of asymmetry for VAS, HHS, THA conversion, adverse events, and WOMAC. No marked asymmetry was observed for OHS, whereas imaging progression and SF-36 were limited by sparse evidence structures. Funnel plot asymmetry was treated as suggestive of small-study effects rather than definitive proof of publication bias, as asymmetry may also arise from clinical heterogeneity, methodological variation, selective outcome reporting, or instability in sparse networks. The presence of funnel plot asymmetry for VAS, HHS, THA conversion, adverse events, and WOMAC suggests that treatment effects may be overestimated in smaller studies (see Figure 6, Supplementary Material 3).

#### 3.4.3 Sensitivity analyses

To evaluate whether the primary NMA results were influenced by study design, follow-up duration, or disease stage, three additional sensitivity analyses were conducted (see Supplementary Figures S1–S11).

RCT-only analysis. When the NMA was restricted to RCTs, treatment rankings for HHS, imaging progression, and THA conversion were generally consistent with the primary analysis. For HHS (14 RCT observations), ESWT-based strategies retained favorable SUCRA rankings, although interpretation remained limited by the inconsistency in the full network. For imaging progression (17 RCT observations), the network was reduced to three connected treatments (placebo, CD, CD plus BMAC); CD plus BMAC maintained the highest probability of being the most favorable intervention. For THA conversion (13 RCT observations), no statistically significant treatment differences emerged. For VAS, the RCT-only network was disconnected because several treatment arms were present only in retrospective studies, precluding a connected NMA.

Follow-up sensitivity analysis. After excluding studies with mean or median follow-up shorter than 24 months, 32 observations remained for imaging progression and 20 for THA conversion. For imaging progression, ESWT-based interventions retained favorable ranking probabilities, and CD plus BMAC remained among the leading active strategies. For THA conversion, no statistically significant differences were detected. These findings suggest that the structural-outcome signals were not driven solely by very short follow-up studies.

Early-stage subgroup analysis. When the analysis was restricted to studies predominantly enrolling patients with early-stage disease (Ficat I–II or ARCO I–II), 26 observations were available for imaging progression and 14 for THA conversion. For imaging progression, ESWT-based interventions and CD plus BMAC remained among the most favorable strategies. For THA conversion, CD plus BMAC and ESWT plus HBO maintained relatively favorable SUCRA probabilities, but no intervention showed statistically significant superiority. This subgroup analysis partly addresses the transitivity concern related to disease stage but does not eliminate residual heterogeneity, as lesion size, lesion location, etiology, and protocol intensity were not uniformly reported. Residual concerns regarding the transitivity assumption therefore persist and should be regarded as an important limitation of the network estimates. Unmeasured differences in key prognostic variables—including lesion size, lesion location, etiology, and treatment intensity—across the included studies represent an ongoing threat to the validity of the indirect comparisons that underpin the network meta-analytical framework, and should be carefully weighed when interpreting treatment rankings.

Taken together, these sensitivity analyses support the general direction of the primary findings. However, the disconnection of the VAS network in the RCT-only analysis highlights the reliance of certain treatment comparisons on retrospective evidence, and the reduced networks in the follow-up and early-stage analyses underscore the need for more adequately powered randomized trials.

## 4 Discussion

This NMA synthesized evidence from 29 comparative studies (2,177 hips) comparing CD, cell therapy, HBO, ESWT, and their combined regimens for ONFH. The main finding was that treatment effects were outcome-specific rather than uniform across endpoints: HBO plus CD ranked favorably for pain relief and quality of life, CD plus ESWT ranked highest for HHS improvement, and CD plus cell therapy together with ESWT showed favorable structural-outcome signals. No intervention demonstrated a statistically significant advantage in reducing THA conversion. These findings are accompanied by substantial between-study heterogeneity, sparse evidence for several outcomes, inconsistency in the HHS and SF-36 networks, and possible small-study effects. Importantly, the adverse-event rankings combine complications of markedly different clinical severity and should be interpreted with corresponding caution; the OHS and SF-36 rankings are preliminary, given that each network included only three studies.

For pain relief and quality of life, HBO-based strategies showed benefits that are consistent with the proposed pathophysiological role of ischemia, bone marrow edema, and impaired local oxygenation in symptomatic ONFH (Uzun et al., 2016; Ansari et al., 2025). Compared with placebo and CD alone, HBO and HBO plus CD showed significant analgesic advantages, with HBO plus CD achieving the highest SUCRA value for VAS. These results extend previous evidence by placing HBO-based therapy within a multi-intervention comparative framework. However, the magnitude of benefit varied across studies, likely reflecting differences in disease stage, treatment duration, HBO protocols (chamber pressure, session frequency and number, adjunctive medications), and follow-up timing. This between-study heterogeneity limits the precision with which an optimal HBO protocol can be defined.

For functional recovery, CD plus ESWT and ESWT-based strategies ranked highest for HHS improvement; ESWT showed a significant advantage over placebo and CD alone. This finding is consistent with evidence that ESWT stimulates angiogenesis, osteogenesis, tissue remodeling, and analgesic pathways in femoral head osteonecrosis (Hao et al., 2018; Sconza et al., 2022; Luan et al., 2022). Importantly, substantial network inconsistency was detected for HHS (loop inconsistency factor, 16.82), indicating that the consistency assumption of NMA may not be fully met for this outcome and that SUCRA-based rankings are therefore inherently uncertain for this outcome. ESWT protocols also varied substantially across studies (energy flux density, number of shocks, number of sessions, imaging guidance, focused versus radial wave technology), which may contribute to outcome heterogeneity.

For structural preservation, CD plus cell therapy and ESWT were associated with lower risks of imaging progression, with the cell-therapy combination ranking first. This result is biologically plausible: CD reduces intraosseous pressure and creates access to the necrotic lesion, while cell therapy may supply progenitor cells, osteogenic precursors, and growth-factor-rich fractions that support osteogenesis and angiogenesis (Andronic et al., 2021; Pawar et al., 2022; Jindal et al., 2021; Hoogervorst et al., 2022). ESWT also ranked highly, suggesting that noninvasive stimulation of bone repair and neovascularization may contribute to structural preservation (Hao et al., 2018; Sconza et al., 2022; Luan et al., 2022; Yang et al., 2022). CD techniques varied across studies (single large-diameter versus multiple small-diameter drilling, with or without grafting), and these differences may independently influence clinical and structural outcomes. Critically, the imaging progression benefit attributed to CD plus BMAC derives from studies that used heterogeneous cell products, preparation methods, and doses, pooled under the BMAC analytical label. The current evidence does not identify which specific cell product, preparation method, or dose is responsible for the observed effect. The reported BMAC effect aggregates biologically distinct cellular therapies—including mesenchymal stem cells, mononuclear cells, cultured stem cells, and osteoblastic-cell preparations—and does not constitute evidence supporting any specific cell product, preparation method, or dose.

THA conversion did not differ significantly among interventions. This contrasts with the more favorable signals for pain, function, and imaging progression and likely reflects the multifactorial nature of THA conversion—it is influenced by baseline stage, lesion size and location, patient age, activity demands, pain tolerance, surgeon preference, local practice patterns, and length of follow-up (Zhao et al., 2020; Pierce et al., 2015; Sultan et al., 2019). Several included studies followed patients for fewer than 24 months, which may be insufficient to capture THA events in early-stage disease. The absence of significant differences may therefore reflect inadequate statistical power and insufficient follow-up rather than true therapeutic equivalence. The sensitivity analysis excluding studies with <24 months of follow-up did not materially alter treatment rankings, but the reduced network limits definitive interpretation.

Regarding evidence stability, imaging progression, THA conversion, and OHS showed no major incoherence between direct and indirect evidence. HHS and SF-36 showed evidence of inconsistency. VAS showed moderate-to-substantial heterogeneity and funnel plot asymmetry. WOMAC was limited to pairwise evidence, showed extreme heterogeneity (I^2^ > 99%), and was sensitive to individual study exclusion. Treatment rankings are therefore most informative when evaluated alongside direct comparison estimates, network geometry, outcome-specific heterogeneity, GRADE certainty ratings (see Table 1), and the clinical characteristics of the included studies. SUCRA probabilities alone do not establish clinically meaningful superiority, particularly when confidence intervals overlap substantially and the evidence base is sparse or heterogeneous.

This study has several clinical contributions. First, treatment selection for ONFH should be outcome-oriented: HBO plus CD may be considered when pain relief and quality-of-life improvement are primary goals; CD plus ESWT and ESWT-based strategies appear promising for functional recovery; CD plus cell therapy and ESWT showed favorable signals for preventing imaging progression. However, the BMAC finding does not identify which specific cell product or protocol is effective, and the optimal regimens for HBO, ESWT, and CD remain to be defined. Second, the findings help distinguish symptomatic, functional, structural, and safety endpoints—these outcomes do not necessarily respond in parallel. Third, the safety rankings warrant careful interpretation: the adverse-event analysis combined complications spanning a wide spectrum of clinical severity—from surgical complications and donor-site morbidity to local discomfort and petechiae—into a single composite endpoint, and the resulting hierarchy does not reflect the differential clinical significance of the component events. Fourth, the analysis provides a comparative framework for discussing treatment options with patients while accounting for disease stage, lesion characteristics, etiology, treatment availability, cost, and patient preference. Fifth, the geographic concentration of evidence—predominantly from China, with smaller contributions from Turkey, Europe, and the United States—limits generalizability to populations with different ONFH etiology distributions, healthcare delivery models, and surgical practice patterns. Sixth, treatment effect estimates and their confidence intervals, rather than SUCRA probabilities alone, should guide clinical interpretation.

Several important limitations should be acknowledged. First, the evidence network was sparse for OHS and SF-36 (three studies each), adverse events, and WOMAC (four studies), reducing precision and ranking stability; findings for these outcomes are preliminary and should be interpreted with caution, as network estimates derived from sparse evidence may be unstable and rankings may shift with the addition of new evidence. Second, substantial inconsistency was observed for HHS and SF-36, indicating that the transitivity assumption of NMA may not be fully met. This inconsistency may reflect differences in disease stage, lesion characteristics, treatment protocols, and outcome measurement timing across studies. Third, the included populations spanned a wide disease-stage spectrum (Ficat I–III, ARCO I–IV, Steinberg I–IV), from early reversible lesions to advanced pre-collapse and post-collapse disease. Pooling across such a broad range may violate the transitivity assumption. Subgroup analyses stratified by disease stage were performed for imaging progression and THA conversion and showed consistent rankings, but could not be performed for symptomatic or functional outcomes (VAS, HHS, OHS, SF-36) due to insufficient data; formal meta-regression was not feasible. Residual concerns regarding the transitivity assumption persist and should be highlighted as an important limitation, because key prognostic variables—including lesion size, lesion location, etiology, and treatment intensity—remain unavailable for quantitative adjustment across studies. Fourth, the sensitivity analysis for structural outcomes excluding studies with <24 months of follow-up showed that treatment rankings were not materially altered, although the reduced networks limit interpretation. ONFH prognosis differs substantially by stage, lesion extent, lesion location, and imaging characteristics; these factors may modify treatment effects (Zhao et al., 2020; Yoon et al., 2020; Pierce et al., 2015). Fifth, different cell-therapy approaches were grouped under the BMAC analytical label, masking clinically important differences in cell source, processing, dose, expansion status, and delivery technique. Pooled effect estimates for CD plus BMAC represent a weighted average across heterogeneous cell products and protocols, not the effect of any single cell-based intervention. The reported BMAC effect aggregates biologically distinct cellular therapies—mesenchymal stem cells, mononuclear cells, cultured stem cells, and osteoblastic-cell preparations—and does not constitute evidence supporting any specific cell product, preparation method, or dose. Protocol-level details, including cell products, preparation methods, and doses, are summarized in Supplementary Material 2 to improve transparency. Sixth, inclusion of retrospective studies increased evidence breadth but introduced potential confounding and selection bias. The RCT-only sensitivity analysis showed consistent treatment rankings for HHS, imaging progression, and THA conversion, although the VAS network became disconnected. Seventh, funnel plot asymmetry and possible small-study effects were observed or could not be excluded for VAS, HHS, THA conversion, adverse events, and WOMAC, suggesting potential overestimation of treatment effects in smaller studies. Eighth, adverse events were classified broadly because systematic stratification by severity, type, or intervention-specific risk profile was not consistently reported across the included studies. The adverse events reported across studies span a wide clinical spectrum—from surgical complications and donor-site morbidity to local discomfort, petechiae, barotrauma, and oxygen toxicity—yet are weighted equally in the network model despite their markedly different clinical significance. The resulting safety rankings should therefore be interpreted with considerable caution, as they do not reflect the differential severity, duration, or intervention-specific risk profiles of the component events. A supplementary table now summarizes the reported adverse events by type and study where information was available. Ninth, the geographic concentration of evidence limits generalizability (Cameron et al., 2015). Tenth, formal meta-regression adjusting for disease stage, follow-up duration, and study design was not feasible and should be revisited when more homogeneous primary studies become available.

Future research should prioritize multicenter RCTs directly comparing the most promising strategies identified here—HBO plus CD, CD plus ESWT, CD plus clearly defined cell-therapy protocols, and ESWT. Trials should use standardized diagnostic and staging criteria; stratify patients by disease stage, lesion size, lesion location, and etiology; describe cellular preparation protocols in detail; and include long-term follow-up sufficient to assess femoral head collapse and THA conversion (Zhao et al., 2020; Yoon et al., 2020; Sultan et al., 2019). Harmonized definitions of imaging progression and adverse events, as well as consistent reporting of patient-reported outcomes (OHS, WOMAC, SF-36), would improve the certainty and clinical applicability of future evidence. Future NMAs should incorporate individual participant data where possible to enable stratification by disease stage, lesion characteristics, and etiology. Mechanistic and biomechanical studies of femoral head collapse, sclerotic-zone stress concentration, microstructural deterioration, and the biological effects of CD may also strengthen interpretation of comparative effectiveness findings and inform more targeted therapeutic strategies (Karasuyama et al., 2015; Chen et al., 2022; Kurup et al., 2025).

## 5 Conclusion

This network meta-analysis indicates that joint-preserving interventions for ONFH have outcome-specific comparative advantages, although the strength of inference varies across endpoints. HBO plus CD showed favorable signals for pain relief and quality of life, although the OHS and SF-36 networks were limited to three studies each and the rankings for HBO-based strategies should be regarded as preliminary. CD plus ESWT showed favorable signals for functional improvement, although substantial network inconsistency (loop inconsistency factor 16.82) limits confidence in the HHS rankings. CD plus cell therapy, as well as ESWT, showed favorable signals for reducing imaging progression. The BMAC benefit represents a pooled estimate across heterogeneous cell products and protocols and does not identify which specific cell product, preparation method, or dose is responsible for the observed effect. No intervention demonstrated significant superiority in reducing THA conversion, which may partly reflect insufficient follow-up and limited statistical power rather than true therapeutic equivalence. The adverse-event analysis combined complications of markedly different clinical severity; the resulting safety rankings should therefore be interpreted with caution and should not be equated with uniform clinical risk across the evaluated interventions. The safety endpoint weighted events ranging from surgical complications to local discomfort equally, and the SUCRA-based hierarchy does not reflect the differential clinical significance of the component events. Residual concerns regarding the transitivity assumption persist because important prognostic variables—including lesion size, lesion location, etiology, and treatment intensity—remain unavailable for quantitative adjustment; these concerns should be carefully weighed when interpreting the network estimates. Treatment selection should be individualized according to disease stage, lesion characteristics, etiology, symptom profile, radiological risk, safety considerations, treatment availability, and patient preference—not based solely on SUCRA probabilities. Confirmation of these comparative signals in dedicated effectiveness trials is warranted. Further high-quality RCTs with standardized protocols, uniform outcome definitions, transparent reporting of cellular preparation methods, stratification by disease stage and etiology, harmonized adverse event reporting, and long-term follow-up (minimum 24 months for structural outcomes) are required to refine the comparative effectiveness hierarchy and guide evidence-based clinical decision-making in ONFH.

## Data Availability

The original contributions presented in the study are included in the article/Supplementary Material, further inquiries can be directed to the corresponding author.
