# Supplementary material for: Comparative efficacy and safety of core decompression, cell-based therapy, hyperbaric oxygen therapy, extracorporeal shock wave therapy, and combined regimens for osteonecrosis of the femoral head: a network meta-analysis
Source: Front Cell Dev Biol. 2026 Jul 15;14:1876711. doi: 10.3389/fcell.2026.1876711 (PMC13416348; doi:10.3389/fcell.2026.1876711)
Supplement: Supplementary file 1 [file Table1.docx]

**Search strategy of PubMed**

| NO. | Search Details | Results |
| --- | --- | --- |
| #8 | (#1 OR #2) AND (#3 OR #4 OR #5 OR #6 OR #7) | 2,316 |
| #7 | ((((Cell and Tissue Based Therapy) OR (Cell Therapy)) OR (Tissue Therapy)) OR (cell based therapy)) OR (cellular therapy) | 1,958,754 |
| #6 | (((((((((((((((Hyperbaric Oxygenations) OR (Hyperbaric Oxygen Therapy)) OR (Hyperbaric Oxygen Therapies)) OR (HBO therapy)) OR (high pressure oxygen)) OR (high tension o2)) OR (high tension oxygen)) OR (hyperbaric medicine)) OR (hyperbaric o2)) OR (hyperbaric oxygen)) OR (hyperbaric oxygen medicine)) OR (hyperbaric oxygen treatment)) OR (hyperbaric oxygenation)) OR (hyperbaric oxygenisation)) OR (hyperbaric oxygenization)) OR (hyperbaric therapy) | 56,342 |
| #5 | (Core Decompression) OR (Decompression) | 72,367 |
| #4 | (((((((((((Extracorporeal Shockwave Therapies) OR (Extracorporeal Shock Wave Therapy)) OR (Shock Wave Therapy)) OR (Shock Wave Therapies)) OR (Extracorporeal High Intensity Focused Ultrasound Therapy)) OR (High Intensity Focused Ultrasound Therapy)) OR (HIFU Therapy)) OR (HIFU Therapies)) OR (extracorporeal shockwave therapy)) OR (shock wave treatment)) OR (shockwave therapy)) OR (shockwave treatment) | 19,058 |
| #3 | (("Extracorporeal Shockwave Therapy"[Mesh]) OR "Hyperbaric Oxygenation"[Mesh]) OR "Cell- and Tissue-Based Therapy"[Mesh] | 352,527 |
| #2 | (((((((((((Femur Head Necroses) OR (Avascular Necrosis of Femur Head)) OR (Ischemic Necrosis Of Femoral Head)) OR (Aseptic Necrosis of Femur Head)) OR (Femoral head necrosis)) OR (femoral head osteonecrosis)) OR (femur head osteonecrosis)) OR (juvenile femur head necrosis)) OR (ONFH)) OR (osteonecrosis of the femoral head)) OR (osteonecrosis of the femur head)) OR (femur head necrosis) | 14,764 |
| #1 | "Femur Head Necrosis"[Mesh] | 9,355 |

**Search strategy of EMBASE**

| No. | Query | Results |
| --- | --- | --- |
| #6 | #1 AND (#2 OR #3 OR #4 OR #5) | 771 |
| #5 | 'core compression' OR 'compression' | 262711 |
| #4 | 'cell therapy'/exp OR 'cell- and tissue-based therapy' OR 'cell and tissue based therapy' OR 'cell therapy' OR 'tissue therapy' OR 'cell based therapy' OR 'cellular therapy' | 389191 |
| #3 | 'hyperbaric oxygen therapy'/exp OR 'hyperbaric oxygenation' OR 'hyperbaric oxygenations' OR 'hyperbaric oxygen therapy' OR 'hyperbaric oxygen therapies' OR 'hbo-therapy' OR 'high pressure oxygen' OR 'high tension o2' OR 'high tension oxygen' OR 'hyperbaric medicine' OR 'hyperbaric o2' OR 'hyperbaric oxygen' OR 'hyperbaric oxygen medicine' OR 'hyperbaric oxygen treatment' OR 'hyperbaric oxygenisation' OR 'hyperbaric oxygenization' OR 'hyperbaric therapy' | 29144 |
| #2 | 'shock wave therapy'/exp OR 'extracorporeal shockwave therapy' OR 'extracorporeal shockwave therapies' OR 'extracorporeal shock wave therapy' OR 'shock wave therapy' OR 'shock wave therapies' OR 'extracorporeal high-intensity focused ultrasound therapy' OR 'extracorporeal high intensity focused ultrasound therapy' OR 'high-intensity focused ultrasound therapy' OR 'high intensity focused ultrasound therapy' OR 'hifu therapy' OR 'hifu therapies' OR 'shock wave treatment' OR 'shockwave therapy' OR 'shockwave treatment' | 7739 |
| #1 | 'femur head necrosis'/exp OR 'femur head necrosis' OR 'femur head necroses' OR 'avascular necrosis of femur head' OR 'ischemic necrosis of femoral head' OR 'aseptic necrosis of femur head' OR 'femoral head necrosis' OR 'femoral head osteonecrosis' OR 'femur head osteonecrosis' OR 'juvenile femur head necrosis' OR 'onfh (osteonecrosis of the femoral head)' OR 'osteonecrosis of the femoral head' OR 'osteonecrosis of the femur head' | 10696 |

**Search strategy of Cochrane Library**

| NO. | Search deatiles | Hits |
| --- | --- | --- |
| #1 | MeSH descriptor: [Cell- and Tissue-Based Therapy] explode all trees | 9462 |
| #2 | MeSH descriptor: [Hyperbaric Oxygenation] explode all trees | 623 |
| #3 | MeSH descriptor: [Extracorporeal Shockwave Therapy] explode all trees | 380 |
| #4 | MeSH descriptor: [Femur Head Necrosis] explode all trees | 203 |
| #5 | ("Cell- and Tissue-Based Therapy" OR "Cell and Tissue Based Therapy" OR "Cell Therapy" OR "Tissue Therapy" OR "cell based therapy" OR "cellular therapy"):ti,ab,kw | 3526 |
| #6 | ("Hyperbaric Oxygenation" OR "Hyperbaric Oxygenations" OR "Hyperbaric Oxygen Therapy" OR "Hyperbaric Oxygen Therapies" OR "HBO-therapy" OR "high pressure oxygen" OR "high tension o2" OR "high tension oxygen" OR "hyperbaric medicine" OR "hyperbaric o2" OR "hyperbaric oxygen" OR "hyperbaric oxygen medicine" OR "hyperbaric oxygen treatment" OR "hyperbaric oxygenisation" OR "hyperbaric oxygenization" OR "hyperbaric therapy"):ti,ab,kw | 1714 |
| #7 | ("Extracorporeal Shockwave Therapy" OR "Extracorporeal Shockwave Therapies" OR "Extracorporeal Shock Wave Therapy" OR "Shock Wave Therapy" OR "Shock Wave Therapies" OR "Extracorporeal High-Intensity Focused Ultrasound Therapy" OR "Extracorporeal High Intensity Focused Ultrasound Therapy" OR "High-Intensity Focused Ultrasound Therapy" OR "High Intensity Focused Ultrasound Therapy" OR "HIFU Therapy" OR "HIFU Therapies" OR "shock wave treatment" OR "shockwave therapy" OR "shockwave treatment"):ti,ab,kw | 2308 |
| #8 | ("Femur Head Necrosis" OR "Femur Head Necroses" OR "Avascular Necrosis of Femur Head" OR "Ischemic Necrosis Of Femoral Head" OR "Aseptic Necrosis of Femur Head" OR "femoral head necrosis" OR "femoral head osteonecrosis" OR "femur head osteonecrosis" OR "juvenile femur head necrosis" OR "ONFH (osteonecrosis of the femoral head)" OR "osteonecrosis of the femoral head" OR "osteonecrosis of the femur head"):ti,ab,kw | 507 |
| #9 | ("Core compression"):ti,ab,kw | 2 |
| #10 | (#1 OR #5 OR #2 OR #6 OR #3 OR #7 OR #9) AND (#4 OR #8) | 76 |

**Search strategy of Web of science**

| NO. | Search deatiles | Hits |
| --- | --- | --- |
| #1 | (TS=("Cell- and Tissue-Based Therapy") OR TS=("Cell and Tissue Based Therapy") OR TS=("Cell Therapy") OR TS=("Tissue Therapy") OR TS=("cell based therapy") OR TS=("cellular therapy")) | 65115 |
| #2 | (TS=("Hyperbaric Oxygenation") OR TS=("Hyperbaric Oxygenations") OR TS=("Hyperbaric Oxygen Therapy") OR TS=("Hyperbaric Oxygen Therapies") OR TS=("HBO-therapy") OR TS=("high pressure oxygen") OR TS=("high tension o2") OR TS=("high tension oxygen") OR TS=("hyperbaric medicine") OR TS=("hyperbaric o2") OR TS=("hyperbaric oxygen") OR TS=("hyperbaric oxygen medicine") OR TS=("hyperbaric oxygen treatment") OR TS=("hyperbaric oxygenisation") OR TS=("hyperbaric oxygenization") OR TS=("hyperbaric therapy")) | 13911 |
| #3 | (TS=("Extracorporeal Shockwave Therapy") OR TS=("Extracorporeal Shockwave Therapies") OR TS=("Extracorporeal Shock Wave Therapy") OR TS=("Shock Wave Therapy") OR TS=("Shock Wave Therapies") OR TS=("Extracorporeal High-Intensity Focused Ultrasound Therapy") OR TS=("Extracorporeal High Intensity Focused Ultrasound Therapy") OR TS=("High-Intensity Focused Ultrasound Therapy") OR TS=("High Intensity Focused Ultrasound Therapy") OR TS=("HIFU Therapy") OR TS=("HIFU Therapies") OR TS=("shock wave treatment") OR TS=("shockwave therapy") OR TS=("shockwave treatment")) | 5944 |
| #4 | (TS=("Femur Head Necrosis") OR TS=("Femur Head Necroses") OR TS=("Avascular Necrosis of Femur Head") OR TS=("Ischemic Necrosis Of Femoral Head") OR TS=("Aseptic Necrosis of Femur Head") OR TS=("femoral head necrosis") OR TS=("femoral head osteonecrosis") OR TS=("femur head osteonecrosis") OR TS=("juvenile femur head necrosis") OR TS=("ONFH (osteonecrosis of the femoral head)") OR TS=("osteonecrosis of the femoral head") OR TS=("osteonecrosis of the femur head")) | 4309 |
| #5 | TS=("Core compression") | 180 |
| #6 | #1 OR #2 OR #3 OR #5 | 84898 |
| #7 | #4 AND #6 | 193 |
