# Supplementary material for: Comparative efficacy and safety of core decompression, cell-based therapy, hyperbaric oxygen therapy, extracorporeal shock wave therapy, and combined regimens for osteonecrosis of the femoral head: a network meta-analysis
Source: Front Cell Dev Biol. 2026 Jul 15;14:1876711. doi: 10.3389/fcell.2026.1876711 (PMC13416348; doi:10.3389/fcell.2026.1876711)
Supplement: Supplementary file 2 [file Table2.docx]

| study | intervention | Details |
| --- | --- | --- |
| Sun M et al(2026) | HBO+CD | The core decompression was performed using the direct lateral approach with a 2 cm skin incision. A fluoroscopic guided large single drill with 8 mm diameter over a guidewire was used to remove the necrotic tissues from the femur head through the lateral cortex distal to the trochanteric tubercle.And the pressure was gradually increased to the target level of approximately 2.2 atmospheres to allow the patient to adjust.Then the patients inhaled 100% oxygen through a tightly sealed, professional-grade face mask to enhance treatment efficacy. |
| Sun M et al(2026) | CD | The core decompression was performed using the direct lateral approach with a 2 cm skin incision. A fluoroscopic guided large single drill with 8 mm diameter over a guidewire was used to remove the necrotic tissues from the femur head through the lateral cortex distal to the trochanteric tubercle. |
| Sun M et al(2026) | HBO | The pressure was gradually increased to the target level of approximately 2.2 atmospheres to allow the patient to adjust.Then the patients inhaled 100% oxygen through a tightly sealed, professional-grade face mask to enhance treatment efficacy. |
| Xu RD et al(2024) | CD + BMAC | Patients underwent spinal or general anesthesia and were positioned supine on a traction table. Under C-arm fluoroscopic guidance, a guide pin was drilled into the femoral head and neck region, ensuring proper placement in anteroposterior and lateral views at the lesion site of femoral head necrosis. A hollow drill was used along the guide pin to create an operational channel. After removing the hollow drill, a decompression sleeve was inserted along the guide pin.A total of 20 μL of bone marrow concentrate was kept for cell counting after the surgery. The average number of bone marrow cells placed to the cylindrical bone was approximately 3 × 109 cells. |
| Xu RD et al(2024) | P | Patients in this group received symptomatic treatment during the therapy period, including using crutches to reduce weight-bearing and taking oral analgesics. They also underwent regular follow-up visits during the follow-up period. |
| Zhu JY et al(2023) | ESWT | Using personalized focused extracorporeal shockwave therapy (fESWT), the shock wave was generated by a focused shock wave generator (HK.SWT-007; Huikan AG, China) with a penetration depth between 0 and 70 mm and a focus diameter of 7.5 mm. |
| Zhu JY et al(2023) | P | 200 mg celecoxib was used once daily for 9 consecutive days. |
| İbrahim Ulusoy et al(2023) | CD + BMAC | Two 30-ml syringes are filled with bone marrow aspirate pre-treated with 5 ml acid citrate dextrose using the appropriate cannula. The sample is then processed using the Angel system according to the manufacturer's instructions. After centrifugation for approximately 17 min, the concentrated content is mixed with 5 ml of demineralised bone matrix to obtain a pasty concentrate. |
| İbrahim Ulusoy et al(2023) | CD | The area corresponding to the area where the measurement was made on the scope was targeted, and the lesion area was reached by entering from the lateral femur with a 2-mm k-wire. A single channel was opened over the K wire with a 10-mm cannulated drill. |
| Liu HJ et al(2023) | CD + ESWT | We repaired damaged cartilage surface and use a 3mm diameter Steiner needle to insert into the subchondral area of the femoral head through the lateral side.And multiple femoral punctures were made through multi-directional drills into the subchondral area of the femoral head. |
| Liu HJ et al(2023) | ESWT | With the necrotic area as the center, the pressure is 0.3Mpa, the impact frequency is 100 times per minute, and the number of impulses is 2,000 times. |
| M. Jayankura et al(2023) | CD + BMAC | PREOB (Bone Therapeutics SA, currently BioSenic SA) is a fresh cell suspension of human autologous bone marrow–derived osteoblastic cells. Bone marrow (between 30 and 50 mL) was harvested from the patient’s hip (iliac crest) under local anesthesia. Bone marrow coagulation was prevented by the addition of heparin. |
| M. Jayankura et al(2023) | CD | Patients underwent fluoroscopically guided core decompression via a small-diameter (5-mm) trephine under general anesthesia |
| Yang X et al（2022） | ESWT | Number of layers, level 3–4(Select the layer according to the patient's feelings. And if the patient feels uncomfortable, it will be reduced to level 3); choose 6 points of the femoral head, each point receives 500 pulses with an energy flux density >0.44 ​mJ/mm2 (level 3); and a total of 3000 pulses with a frequency of 2 ​Hz. Each treatment time is about 25–30 ​min. |
| Yang X et al（2022） | P | All cases in group B did not receive shock wave intervention. Alendronate sodium tablets |
| Wang QR et al(2022) | CD | Patients underwent surgery in the supine position under general anesthesia. A guide pin was inserted into the lateral cortex of the femur and directed towards the proximal part of the femoral neck. The alignment of the guide pin was checked under C‐arm fluoroscopy guidance to ensure that the pin was centered in the femoral head at the appropriate depth. Then the guide pin was removed and a Steinmann pin with a diameter of 4 mm was advanced into the lesion under fluoroscopic guidance. |
| Wang QR et al(2022) | P | No surgical treatment |
| Shi LJ et al(2022) | ESWT | ESWT group received two sessions of ESWT with oral drug treatment. |
| Shi LJ et al(2022) | P | Patients in the control group received oral drug treatment only. |
| I.Bozkurt et al(2022) | HBO+CD | Hip decompression was done under fluoroscopic guidance in two planes. After determination of the entry point, an approximately 2–4 cm incision was made over the lateral aspect of the thigh. The guide wire was then placed into the centre of the previously identified necrotic lesion, typically in the anterosuperior region of the femoral head, under fluoroscopic control. |
| I.Bozkurt et al(2022) | HBO | Hyperbaric oxygen therapy was solely applied or com bined with CD 1 week after the operation. All patients underwent sessions in a multiplace pressure chamber (Haux Starmed 2200/5,5; Haux Life Support GmbH, Karlsbad-Ittersbach Germany). The sessions were applied 6 days a week, at 2.4 ATA for 120 min, for a total of 30 ses sions in each group |
| Y. Tomaru et al(2021) | CD + BMAC | CABMAT was performed according to an established protocol. Approximately 300 mL of bone marrow was aspirated from the iliac crest using a bone marrow harvest needle (Medical Device Technologies, Inc., Gainesville, FL, USA) and transferred into a bone marrow collection bag (Baxter, Deerfield, IL, USA). After the centrifugation at 1,200 g for 10 min (KUBOTA 9800; Kubota, Osaka, Japan), erythrocytes were transferred from the main bag to a satellite bag (Terumo, Tokyo, Japan). After the second centrifugation (3,870 g for 7 min), the plasma was transferred from the main bag into another satellite bag; therefore, approximately 30 mL of the buffy coat was left in the main bag. Core decompression using a 4.8-mm-diameter trephine (Iso Medical Systems, Tokyo, Japan) was performed to transport the buffy coat to the necrotic area. The drill was inserted percutaneously into the center of the necrotic area. After the core decompression, multiple drillings toward the necrotic site from the core decompression hole were performed using 2.4-mm-diameter Kirschner wires. Using the core decompression tract, a cannulated metal rod with four small holes at the top (Iso Medical Japan, Tokyo, Japan) was inserted into the necrotic area. Through the metal rod, the buffy coat was manually inserted and slowly infused over several minutes using a syringe. Approximately 18 ± 5.7 mL of the buffy coat was transplanted using a monitor with biplane fluoroscopy. |
| Y. Tomaru et al(2021) | P | Patients are treated conservatively |
| I. Moghamis et al(2021) | CD | All patients were positioned in a supine position on a fracture table. The core decompression was performed using the direct lateral approach with a 2 cm skin incision. A fluoroscopic guided large single drill with 8 mm diameter over a guidewire was used to remove the necrotic tissues from the femur head through the lateral cortex distal to the trochanteric tubercle. After the removal of the core necrotic bone, the track was filled with a 5 ml synthetic bone paste substitute. |
| I. Moghamis et al(2021) | HBO | After optimization of patients, each patient had received between 25 and 40 sessions of HBO therapy, with 3–4 sessions per week to avoid any possibility of oxygen toxicity. The treatment protocol was delivered by a HBO therapy specialist and involved breathing 100% oxygen at 22.5 pounds per square inch (2.2 atm) in a hyperbaric oxygen pressure chamber for 90 min. With additional 15 min for decompression till reaching 22.5 pounds per square inch, two air breaks of 5 min and 15 min for recompression back to surface. Each patient was provided with a well-sealed breathing mask from which he or she received the oxygen treatment. |
| P. Martinot et al(2020) | CD | The CD was performed by a small incision on the lateral thigh to make a single hole in the lateral cortex with a small manual trephine (4.3 mm). Under fluoro scopic guidance, a pin was used to reach the necrosis area and several passages were made to stimulate revascularization of the entire necrotic part of the femoral head. |
| P. Martinot et al(2020) | CD + BMAC | A marrow aspiration from the ipsilateral iliac crest was performed, and approximately 20 cc of non-concentrated bone marrow was reinjected via a cannula inserted into the previously drilled hole. |
| Li M et al(2020) | CD | For core decompression of the femoral head, we first determined the optimal entrance point for drilling, and then a 1.5-cm incision was made at the level of the greater trochanter. A 3.0-mm-diameter Kirschner wire (K-wire) was introduced into the necrotic area with the tip placed in the subchondral bone area approximately 2 to 3 mm from the articular cartilage. Next, a 10-mm-diameter trephine was drilled through the K-wire to the necrotic region. A cylinder of bone from the femoral neck and head was obtained. |
| Li M et al(2020) | CD + BMAC | The bone marrow was centrifuged at 1500 rpm for 10 min in a bench-top centrifuge (Eppendorf, AG 22331, Hamburg, Germany) with a sterilized chamber. The bone marrow was separated into three phases after centrifugation. We collected a total of 1 mL of bone marrow concentrate from the interface containing enriched bone marrow cells with a sterilized transfer pipette, and then, the bone marrow concentrate was seeded on the cylindrical bone drop by drop to allow the cells to anchor onto the bone surface. A total of 10 μL of bone marrow concentrate was kept for cell counting after the surgery. The average number of bone marrow cells loaded onto the cylindrical bone was approximately 3 × 109 nucleated cells. |
| F. J. Nally et al(2018) | CD | CD was performed through perforations with cannulated trephine of 5mm from the lateral aspect of the femur in the necrosis affected area. |
| F. J. Nally et al(2018) | CD + BMAC | The sample was placed in sterile Ficoll-Paque Premium(3ml) to isolate stem cells in the interface. |
| J. S. Kang et al(2018) | CD | All patients underwent surgery under spinal anesthesia. Guided by a fluoroscopic view through the greater trochanter and the femoral neck, using 2.0-mm K-wires, core decompression tunnels were made into the subchondral necrotic lesion of the femoral head (2–3 mm away from the joint cartilage). The centrally positioned K-wire was over drilled using trephine. Subsequently, the necrotic tissues were removed from the femoral head. |
| J. S. Kang et al(2018) | CD + BMAC | The bone marrow was aspirated up to 100–120 mL through the decompression tunnel at the subtrochanteric, proximal femur area, and diluted with the same amounts of Hanks’ balanced salt solution (HBSS; GIBCO-BRL, Grand Island, NY, USA). Then, Ficoll-Paque Plus (1077 g/L; Amersham Biosciences, Piscataway, NJ, USA) solution was added in the same ratio. Centrifugation for 30 min at 1000g separated the mononuclear cells from the remainder of the marrow. The mononuclear cells were then collected and washed with HBSS three times before another 15-min centrifugation at 900g. Subsequently, the mononucleocyte layer was collected and 1.8 mL of phosphate buffered saline (Gibco, Carlsbad, CA, USA) was added to suspend the cells. The mean injected leukocyte cell count and average stem cells per colony-forming units (CFUs) were not calculated for all patients. However, in 17 hips in 16 patients, the cell counts were confirmed by a pilot study. The average density of the autologous marrow monocytes was 13.97 ± 13.8 × 106/mL (range from 1.37 × 106/mL to 59.6 × 106/mL). The mean number of fibroblast CFUs per million nucleated cells obtained from each patient was 21.6 ± 11.01 (range from 7 to 53). |
| P. Hernigou et al(2018) | CD | The trocard was introduced through the trochanter, as in conventional core decompression with a percutaneous approach using a 4-mm diameter trocard (the same trocard as for bone marrow injection). Its position in the femoral head and in the necrotic segment was monitored with fluoroscopy. The direction of the trocard was adjusted in both planes with anteroposterior and lateral fluoroscopy view. The position of the tip was directed into the osteonecrosis at a distance of 5 mmfrom the articular cartilage. |
| P. Hernigou et al(2018) | CD+BMAC | For hips with bone marrow injection, the number of mononucleated cells per ml of BM was 25.8±8 million in the initial bone marrow aspirate and increased after concentration to 104 ± 25 cells/mL. After ten days in culture, an average of 28.3± 19.9 CFU-F colonies per million of mononucleated cells was observed in BM aspirates cultures. Each femoral head received 20 ml of bone marrow whatever the size of the necrotic lesion. The average total number of MSCs (counted as CFU-F) injected in each hip was90,000±25,000cells (range 45,000to180,000 cells). |
| J. P. Hauzeur et al(2018) | CD | It was performed by a small incision on the lateral thigh to make a single hole in the lateral cortex with a small manual trephine. |
| J. P. Hauzeur et al(2018) | CD + BMAC | Under general anaesthesia, a volume of 400 mL of bone marrow was harvested from the posterior iliac crests and was sorted on a Spectra cell separator (777,006,300; Cobe, Lakewood,Colorado) and concentrated to a final volume of 50ml. During the same general anaesthesia, a special 4 mm trephine was inserted under control of a fluoroscopic view through the great trochanter, the neck, and the femoral head in the necrotic lesion. |
| H. Pilge et al(2016) | CD + BMAC | A total of 60 mL of BMA was extracted with 20 mL syringes, which were pre-flushed with heparin. |
| H. Pilge et al(2016) | CD | A small lateral approach to the femur was performed; a decompression tunnel was made using a trephine through the lateral femur and femoral neck into the necrotic lesion in the subchondral area. |
| A. L. Gianakos et al(2016) | CD + BMAC | All syringes had been previously treated with a solution of saline and heparin to avoid clotting. The aspiration was then concentrated into about 15 ml of the bone marrow cells using the bone marrow aspiration system (Harvest Technologies Corporation, Plymouth, MA). |
| A. L. Gianakos et al(2016) | P | If patient symptoms did not resolve and vitamin D levels were normal, the patients were then prescribed 35 mg of Actonel (bisphosphonate) taken orally once a week with follow-up visits every month. |
| Yan Dl et al(2015) | CD | All patients were placed prone on a radiolucent operating table with both hip joints in neutral rotation and were given general anesthesia. Bone marrow was obtained from the ipsilateral iliac crest as previously reported.16 Core decompression was performed using a 4.5-mm diameter drill. |
| Yan Dl et al(2015) | CD + BMAC | In the MSC group, at the time of bone marrow harvest MSCs were obtained by centrifugation and filtration.16 After core decompression, the concentrated MSCs were injected through the tunnel, which opened during core decompression (Figure 1).The entrance of the tunnel was closed with biomaterials(Osteoset, Wright, USA) to prevent leakage of the concentrated cells. Low-molecular-weight heparin was prescribed to all patients for thromboembolism prophylaxis for 3 weeks. |
| Wang CJ et al(2015) | ESWT | Shock waves were administered with an electrohydraulic OssaTron orthotriptor (High Medical Technology, Kreuzlingen,Switzerland) in one treatment, performed with the patient under general anesthesia and in the supine position. The affected hip was positioned in adduction and internal rotation with the limb secured to the table. |
| Wang CJ et al(2015) | CD | Surgery was performed through a lateral approach with the patient on a fracture table and under either general or spinal anesthesia. The location of the osteonecrosis of the femoral head was verified with c-arm imaging. |
| R. M. Tabatabaee et al(2015) | CD + BMAC | The aspirated bone marrow was filtered and washed to remove fat,clotdebris, and redblood cells(RBCs).The bone marrow was centrifuged for 5–10min at 400g.The heavier polynuclear cells were removed from the periphery at a flowrate of 100 ml/min for about 50 seconds. The lighter anucleated RBCs and plasma were collected from the center.If the mean MNC count per ml wasat least 2 million cells, the concentrated MNCs were sent back to the operation room in a sterile plastic bag containing anticoagulants for injection. The processing of the bone marrow aspirate took place in less than 60 minutes. |
| R. M. Tabatabaee et al(2015) | CD | Core decompression was done using the classic technique. After the preparationoftheskinasmallincisionwasmadeonthelateralaspectof the hip, just below the greater trochanter. The lateral cortex was drilled using a 2.7 mm drill, and the drill advanced into the necrotic aspect of the femoral head under floursocopy. |
| Ma YC et al(2014) | CD | The patients were positioned supine on the operation table and anesthetized by continuous epidural anesthesia. For core decompression of the femoral head, a 1.5-cm incision was made over the skin and the fascia on the lateral aspect the thigh at the level of the greater trochanter. |
| Ma YC et al(2014) | CD + BMAC | To obtain the BBC with enriched nucleated cells, bone marrow was centrifuged at 1500 revolutions per minute for 10 minutes in a bench-top centrifuge with a sterilized chamber. After centrifuge, the bone marrow was separated into three phases. The blood serum from the superficial layer was removed, and the BBC from the interface containing enriched bone marrow cells was collected by a sterilized transfer pippet and carefully loaded onto the porous cylindrical bone drop by drop. A total of 1 mL of BBC was isolated from the suspension, and 10 μL of BBC was kept for cell counting after the surgery. The average bone marrow cells loaded to the cylindrical bone were approximately 3 × 109 nucleated cells. |
| Shan-Ling Hsu et al(2010) | ESWT+HBO | HBO was performed while patients were in a sealed multi-place chamber at a pressure of 2.5 atmospheres absolute (ATA). The air pressure was gradually increased from 1 to 2.5 ATA in 15min. 100% medical grade oxygen was inhaled through a plastic facemask for 2 periods of 25min each with a 5min break in between, for a total of 90min per treatment. The air pressure was then decompressed from 2.5 ATA down 1.0 ATA within 15min to complete the treatment. HBO was performed once a day,5 times a week for 4weeks, for a total of 20 treatments. |
| Shan-Ling Hsu et al(2010) | ESWT | Each affected hip was treated with 6,000 shocks of ESWT at 28 kV(equivalent to 0.62 mJ/mm2) in a single session. |
| Enrico M. Camporesi et al(2010) | HBO | Patients were exposed to HBO inside a multiplace hyperbaric chamber (Galeazzi, Bergamo, Italy) with either compressed oxygen or compressed air at 2.5 ATA for 82 minutes, comprising a period of 60 minutes when the patient was continuously exposed to 2.5 ATA without interruption. Each patient was provided with a well sealed breathing mask from which he or she received either 100% oxygen (HBO) or 100% compressed air(HBA). |
| Enrico M. Camporesi et al(2010) | P | Patients were exposed to HBO inside a multiplace hyperbaric chamber (Galeazzi, Bergamo, Italy) with either compressed oxygen or compressed air at 2.5 ATA for 82 minutes, comprising a period of 60 minutes when the patient was continuously exposed to 2.5 ATA without interruption. Each patient was provided with a well sealed breathing mask from which he or she received either 100% oxygen (HBO) or 100% compressed air(HBA). |
| Ramesh Kumar Sen et al(2012) | CD | Under the guidance of image intensifier, 3 cores of 4mm diameter were drilled from the lateral cortex to the site of lesion.Core biopsy was taken from all hips for histologic confirmation of osteonecrosis. |
| Ramesh Kumar Sen et al(2012) | CD + BMAC | Under epidural anesthesia,nearly 120 to 180mL of bone marrow was aspirated from the posterior iliac crest with 10-mL syringe, each time changing the site or direction of aspiration.Thea spirate was processed in the laboratory (by Ficoll interface separator) until the mononuclearcell concentrate(MNC,2mL) was available. |
| Zhao DW et al(2012) | CD | The procedures for CD treatment including establishment of the decompression tunnel and removal of the necrotic segment from the femoral head were identical to the BMMSC treatment as described above. |
| Zhao DW et al(2012) | CD+BMAC | After 10 mL of subtrochanteric bone marrow was aspirated through the decompression tunnel, the necrotic segment was removed by a custom-made trephine with a collapsible scraping end (Fig. 2E). Next, the bored bone core was plugged into the decompression tunnel before the outlet of the decompression tunnel was sealed with bone wax followed by layer closure. The subtrochanteric bone marrow-derived BMMSCs were subjected to proliferation in vitro for two weeks, after which about 2×106 BMMSCs were harvested and prepared in 2ml normal saline solution later injected into the osteonecrotic site in 3 weeks following the operation. Patients were then instructed to practice weight bearing with a maximum of 30% body weight 4 weeks post-operation. Full weight bearing was permitted at the beginning of the 6th week post-operation. |
| V.Gangji et al(2011) | CD | However,core decompression of the hip with the use of an 8-mm trephine is still the most common procedure used to treat early stages of the disease. In this study,a 3mm trephine had to be used to avoid leakage of the implanted bone marrow. |
| V.Gangji et al(2011) | CD + BMAC | The bone marrow harvest from the iliac crest was sorted and concentrated to a final volume of 49.7±2.3 ml. The sorted bone marrow contained 1.9±0.2×109 mononuclear cells including 1.0±0.1% of CD34+ cells which are precursors of hematopoietic cells and 92.6±22.4×107/cells of fibroblast colony-forming units,an indicator of stromal cell activity. |
