# Supplementary material for: Comparative efficacy and safety of core decompression, cell-based therapy, hyperbaric oxygen therapy, extracorporeal shock wave therapy, and combined regimens for osteonecrosis of the femoral head: a network meta-analysis
Source: Front Cell Dev Biol. 2026 Jul 15;14:1876711. doi: 10.3389/fcell.2026.1876711 (PMC13416348; doi:10.3389/fcell.2026.1876711)
Supplement: Supplementary file 3 [file Table5.docx]

**Supplementary Appendix5: Consistency, Heterogeneity, and Sensitivity Assessments**

This appendix summarizes the inconsistency diagnostics for network meta-analysis outcomes and the additional conventional meta-analysis diagnostics for WOMAC. Network outcomes were assessed using loop-specific inconsistency factors or ratio of odds ratios, global inconsistency tests, and node-splitting models. WOMAC was evaluated in a conventional pairwise meta-analysis framework; therefore, its Galbraith plot and leave-one-out sensitivity results are reported separately rather than pooled with network inconsistency diagnostics.

For ROR-based loop inconsistency, the null value is 1; for IF-based loop inconsistency, the null value is 0. P < 0.05 was considered suggestive of inconsistency. Treatment-code labels were retained from the original Stata output to preserve traceability and should be mapped to the treatment definitions in the network geometry figure or supplementary legend.

**Network meta-analysis inconsistency diagnostics**

**Supplementary Table S1. Loop-specific heterogeneity and inconsistency assessment across network outcomes.**

| **Outcome** | **Closed loop** | **Measure** | **Estimate** | **95% CI** | **Loop-specific heterogeneity (tau^2)** | **Interpretation** |
| --- | --- | --- | --- | --- | --- | --- |
| VAS | CD-CD+BMAC-ESWT-Placebo | IF | 2.31 | 0.00 to 7.04 | 2.557 | No statistically evident loop inconsistency; CI includes 0. |
| HHS | CD-CD+BMAC-ESWT-Placebo | IF | 16.82 | 2.26 to 31.37 | 17.307 | Statistically evident loop inconsistency; CI excludes 0. |
| Imaging progression | CD-HBO-HBO+CD | ROR | 2.540 | 1.00 to 56.02 | 0.000 | No clear loop inconsistency; interval includes the null value of 1. |
| Imaging progression | CD-Placebo-ESWT | ROR | 2.417 | 1.00 to 22.68 | 0.000 | No clear loop inconsistency; interval includes the null value of 1. |
| THA conversion | CD-CD+BMAC-Placebo | ROR | 1.335 | 1.00 to 36.13 | 1.758 | No clear loop inconsistency; interval includes the null value of 1. |
| OHS | CD-HBO-HBO+CD | IF | 1.10 | 0.00 to 6.79 | 0.000 | No clear loop inconsistency; interval includes the null value of 0. |
| SF-36 | CD-HBO-HBO+CD | IF | 0.52 | 0.00 to 9.27 | 0.000 | No clear loop inconsistency; CI includes the null value of 0. |

Abbreviations: BMAC, bone marrow aspirate concentrate; CD, core decompression; CI, confidence interval; ESWT, extracorporeal shock wave therapy; HBO, hyperbaric oxygen; HHS, Harris Hip Score; IF, inconsistency factor; OHS, Oxford Hip Score; ROR, ratio of odds ratios; SF-36, 36-item Short Form Health Survey; THA, total hip arthroplasty; VAS, visual analogue scale.

**Supplementary Table S2. Global inconsistency tests based on the multivariate meta-analysis model.**

| **Outcome** | **Constraint(s) tested** | **chi-square** | **df** | **P value** | **Conclusion** |
| --- | --- | --- | --- | --- | --- |
| VAS | [y_B]des_BD = 0 | 0.94 | 1 | 0.3322 | No statistically significant inconsistency. |
| HHS | [y_B]des_BE = 0 | 7.96 | 1 | 0.0048 | Statistically significant inconsistency. |
| Imaging progression | [y_B]des_BD = 0; [y_F]des_BFG = 0 | 0.65 | 2 | 0.7213 | No statistically significant global inconsistency. |
| THA conversion | [y_C]des_BC = 0 | 0.06 | 1 | 0.8038 | No statistically significant global inconsistency. |
| OHS | [y_C]des_BCD = 0 | 0.16 | 1 | 0.6896 | No statistically significant global inconsistency. |
| SF-36 | [y_B]des_ABC = 0; [y_C]des_BC = 0 | 8.62 | 2 | 0.0135 | Statistically significant global inconsistency. |

Global tests were interpreted at the two-sided 0.05 significance level. Statistically significant findings indicate evidence against the consistency assumption for the corresponding outcome network.

**Supplementary Table S3. Node-splitting analysis for VAS.**

| **Side** | **Direct estimate** | **Direct SE** | **Indirect estimate** | **Indirect SE** | **Difference** | **Difference SE** | **P value** | **tau** | **Interpretation** |
| --- | --- | --- | --- | --- | --- | --- | --- | --- | --- |
| A-C | -1.292352 | 1.147951 | 1.018713 | 2.089772 | -2.311065 | 2.384340 | 0.332 | 1.580504 | No inconsistency |
| A-D | -0.740124 | 1.127697 | -3.052082 | 2.101039 | 2.311958 | 2.384536 | 0.332 | 1.580518 | No inconsistency |
| A-F* | -3.789367 | 1.781843 | -0.000004 | 316.229200 | -3.789363 | 316.234200 | 0.990 | 1.579063 | Unstable indirect estimate |
| B-C | -1.962941 | 1.579696 | 0.474807 | 2.312190 | -2.311866 | 2.384628 | 0.332 | 1.580524 | No inconsistency |
| B-D | -3.722840 | 1.661715 | -1.410613 | 1.710319 | -2.312270 | 2.384636 | 0.332 | 1.580524 | No inconsistency |
| D-E* | 0.283143 | 1.592074 | 2.803633 | 633.603700 | -2.520489 | 633.605600 | 0.997 | 1.579062 | Unstable indirect estimate |
| D-H* | -0.922549 | 1.592284 | 1.592106 | 631.407200 | -2.514656 | 631.409100 | 0.997 | 1.579059 | Unstable indirect estimate |
| F-G* | -1.112381 | 1.597753 | 6.466497 | 633.281800 | -7.578878 | 633.283700 | 0.990 | 1.579063 | Unstable indirect estimate |

Asterisk indicates contrasts for which the original Stata output warned that all evidence came from trials directly comparing the interventions. Very large indirect SEs indicate weakly informed indirect estimates.

**Supplementary Table S4. Node-splitting analysis for HHS.**

| **Side** | **Direct estimate** | **Direct SE** | **Indirect estimate** | **Indirect SE** | **Difference** | **Difference SE** | **P value** | **tau** | **Interpretation** |
| --- | --- | --- | --- | --- | --- | --- | --- | --- | --- |
| A-C | 9.692061 | 4.017980 | -7.944870 | 4.817546 | 17.636930 | 6.257951 | 0.005 | 3.265968 | Significant inconsistency |
| A-F | 5.653169 | 2.823713 | 23.291180 | 5.610004 | -17.638010 | 6.258293 | 0.005 | 3.267330 | Significant inconsistency |
| B-C | 7.081231 | 1.623912 | 24.736460 | 6.040093 | -17.655230 | 6.258149 | 0.005 | 3.266081 | Significant inconsistency |
| B-E | 20.699940 | 3.555928 | 3.044222 | 5.148700 | 17.655710 | 6.257304 | 0.005 | 3.266595 | Significant inconsistency |
| D-E* | -3.860001 | 5.632720 | 21.526580 | 632.567300 | -25.386580 | 632.594300 | 0.968 | 5.527210 | Unstable indirect estimate |
| E-F* | -3.000000 | 5.952174 | -20.665470 | 632.633600 | 17.665470 | 632.653800 | 0.978 | 5.527233 | Unstable indirect estimate |

Asterisk indicates contrasts for which the original Stata output warned that all evidence came from trials directly comparing the interventions. Very large indirect SEs indicate weakly informed indirect estimates.

**Supplementary Table S5. Node-splitting analysis for imaging progression.**

| **Side** | **Direct estimate** | **Direct SE** | **Indirect estimate** | **Indirect SE** | **Difference** | **Difference SE** | **P value** | **tau** | **Interpretation** |
| --- | --- | --- | --- | --- | --- | --- | --- | --- | --- |
| A-B | -0.8711491 | 0.7565169 | 0.0112820 | 1.426086 | -0.8824310 | 1.614323 | 0.585 | 0.658 | No statistically significant disagreement. |
| A-D | -1.5604300 | 1.0358720 | -2.4427620 | 1.238283 | 0.8823321 | 1.614427 | 0.585 | 0.658 | No statistically significant disagreement. |
| B-C* | -1.3943530 | 0.3544297 | -0.0234531 | 260.4698 | -1.3709000 | 260.4697 | 0.996 | 0.610 | Indirect estimate weakly informed. |
| B-D | -1.5716990 | 0.9803464 | -0.6893033 | 1.282699 | -0.8823954 | 1.614434 | 0.585 | 0.658 | No statistically significant disagreement. |
| B-F* | -0.1299747 | 0.7275439 | 1.2382160 | 451.5436 | -1.3681910 | 451.5434 | 0.998 | 0.616 | Indirect estimate weakly informed. |
| B-G* | 1.0986080 | 1.1119300 | -0.7654029 | 2.859372 | 1.8640110 | 2.972306 | 0.531 | 0.643 | No statistically significant disagreement. |
| D-F* | -0.0408220 | 0.9057346 | 3.8127860 | 636.4500 | -3.8536080 | 636.4499 | 0.995 | 0.616 | Indirect estimate weakly informed. |
| F-G* | 0.8109302 | 1.1323530 | 2.6750080 | 2.835252 | -1.8640780 | 2.972333 | 0.531 | 0.643 | No statistically significant disagreement. |

Asterisk indicates contrasts for which the original Stata output warned that all evidence came from trials directly comparing the interventions. Very large indirect SEs indicate weakly informed indirect estimates.

**Supplementary Table S6. Node-splitting analysis for THA conversion.**

| **Side** | **Direct estimate** | **Direct SE** | **Indirect estimate** | **Indirect SE** | **Difference** | **Difference SE** | **P value** | **tau** | **Interpretation** |
| --- | --- | --- | --- | --- | --- | --- | --- | --- | --- |
| A-B | -0.8175623 | 1.1278010 | -1.1622490 | 0.8103365 | 0.3446868 | 1.388733 | 0.804 | 1.060 | No statistically significant disagreement. |
| A-C | -1.3698110 | 0.7036471 | -1.0248600 | 1.1986080 | -0.3449510 | 1.388653 | 0.804 | 1.060 | No statistically significant disagreement. |
| B-C | -0.2074627 | 0.4061862 | -0.5523302 | 1.3292860 | 0.3448675 | 1.388725 | 0.804 | 1.060 | No statistically significant disagreement. |
| B-D* | -1.4122690 | 1.2443850 | 0.9814079 | 282.7375 | -2.3936770 | 282.7399 | 0.993 | 1.000 | Indirect estimate weakly informed. |
| D-E* | -0.0408220 | 1.2057320 | 4.8869520 | 631.8003 | -4.9277740 | 631.8008 | 0.994 | 1.000 | Indirect estimate weakly informed. |

Asterisk indicates contrasts for which the original Stata output warned that all evidence came from trials directly comparing the interventions. Very large indirect SEs indicate weakly informed indirect estimates.

**Supplementary Table S7. Node-splitting analysis for OHS.**

| **Side** | **Direct estimate** | **Direct SE** | **Indirect estimate** | **Indirect SE** | **Difference** | **Difference SE** | **P value** | **tau** | **Interpretation** |
| --- | --- | --- | --- | --- | --- | --- | --- | --- | --- |
| A-B* | 5.2999990 | 1.2658590 | 0.0000388 | 223.6544 | 5.299960 | 223.6579 | 0.981 | 8.730 | Indirect estimate weakly informed. |
| B-C* | 0.5853761 | 1.0914370 | -10.0146000 | 450.5498 | 10.599980 | 450.5485 | 0.981 | 3.870 | Indirect estimate weakly informed. |
| B-D* | 2.5500330 | 1.1759570 | 0.3491945 | 5.281747 | 2.200839 | 5.511870 | 0.690 | 0.003 | No statistically significant disagreement. |
| C-D* | 1.7500090 | 0.9627635 | 3.9499930 | 5.413662 | -2.199985 | 5.516870 | 0.690 | 0.008 | No statistically significant disagreement. |

Asterisk indicates contrasts for which the original Stata output warned that all evidence came from trials directly comparing the interventions. Very large indirect SEs indicate weakly informed indirect estimates.

**Supplementary Table S8. Node-splitting analysis for SF-36.**

| **Side** | **Direct estimate** | **Direct SE** | **Indirect estimate** | **Indirect SE** | **Difference** | **Difference SE** | **P value** | **tau** | **Interpretation** |
| --- | --- | --- | --- | --- | --- | --- | --- | --- | --- |
| A-B* | 2.876083 | 1.400245 | -16.15237 | 6.376153 | 19.02845 | 6.491678 | 0.003 | 2.27 | Significant direct-indirect disagreement. |
| A-C | 3.610023 | 3.997141 | 13.37678 | 7.478807 | -9.766758 | 8.488235 | 0.250 | 3.79 | No statistically significant disagreement. |
| B-C* | 5.105923 | 4.791646 | 1.982791 | 18.8964 | 3.123132 | 19.50952 | 0.873 | 6.34 | No statistically significant disagreement; indirect estimate imprecise. |

Asterisk indicates contrasts for which the original Stata output warned that all evidence came from trials directly comparing the interventions. Very large indirect SEs indicate weakly informed indirect estimates. The A-B contrast showed statistically significant direct-indirect disagreement (P = 0.003).

**Conventional pairwise meta-analysis diagnostics for WOMAC**

Because WOMAC was analyzed using a conventional pairwise random-effects meta-analysis rather than a connected treatment network, node-splitting, loop-specific, and global network inconsistency diagnostics were not applicable. The available diagnostic evidence is therefore summarized separately using the Galbraith plot and leave-one-out sensitivity analysis.

**Supplementary Table S9. Galbraith plot assessment for WOMAC.**

| **Outcome** | **Diagnostic method** | **Model/information available** | **Key finding** | **Interpretation** |
| --- | --- | --- | --- | --- |
| WOMAC | Galbraith radial plot | Four studies; random-effects variance incorporated in the SE definition in the plot | No study was visually located outside the 95% confidence limits. | No obvious outlier signal was detected by visual Galbraith assessment. |

The Galbraith plot is presented as a visual diagnostic. Formal numerical outlier statistics were not provided in the submitted output.

**Supplementary Table S10. Leave-one-out sensitivity analysis for WOMAC under a random-effects DerSimonian-Laird model.**

| **Outcome** | **Omitted study** | **Pooled mean difference** | **95% CI** | **P value** | **Interpretation** |
| --- | --- | --- | --- | --- | --- |
| WOMAC | Li M et al. (2020) | 8.83 | -1.75 to 19.41 | 0.102 | The pooled effect was not statistically significant after omission. |
| WOMAC | J. P. Hauzeur et al. (2018) | 16.67 | 8.75 to 24.60 | <0.001 | The pooled effect remained statistically significant after omission. |
| WOMAC | R. M. Tabatabaee et al. (2015) | 10.85 | 1.02 to 20.69 | 0.031 | The pooled effect remained statistically significant after omission. |
| WOMAC | Ma YC et al. (2014) | 13.97 | -0.83 to 28.77 | 0.064 | The pooled effect became borderline/non-significant after omission. |

Values are mean differences with 95% confidence intervals. The sensitivity profile suggests that the direction of effect was generally preserved, although statistical significance depended on which study was omitted.

Reporting note: These supplementary tables are intended for appendix use. Before final submission, treatment-code labels should be checked against the network geometry and all outcome labels should be harmonized with the terminology used in the main manuscript.
