# Supplementary material for: Comparative efficacy and safety of core decompression, cell-based therapy, hyperbaric oxygen therapy, extracorporeal shock wave therapy, and combined regimens for osteonecrosis of the femoral head: a network meta-analysis
Source: Front Cell Dev Biol. 2026 Jul 15;14:1876711. doi: 10.3389/fcell.2026.1876711 (PMC13416348; doi:10.3389/fcell.2026.1876711)
Supplement: Supplementary file 4 [file Table3.docx]

**Supplementary Table S1-S7: Network Meta-analysis League Tables**

**General note.** The following league tables are presented in the order specified for the manuscript: VAS, HHS, imaging progression evaluation, THA conversion, OHS, SF-36, and adverse events. Each cell shows the effect estimate with 95% confidence interval (CI) for the column-defined intervention compared with the row-defined intervention.Bold values indicate that the 95% CI excludes the null value.

**Abbreviations:** BMAC denotes the pooled analytical category for cell therapy; CD, core decompression; CI, confidence interval; ESWT, extracorporeal shock wave therapy; HBO, hyperbaric oxygen therapy; HHS, Harris Hip Score; OHS, Oxford Hip Score; SF-36, 36-item Short Form Health Survey; THA, total hip arthroplasty; VAS, visual analogue scale.

**Supplementary Table S1. League table for visual analogue scale (VAS).**

| **Placebo** |  |  |  |  |  |  |  |
| --- | --- | --- | --- | --- | --- | --- | --- |
| 2.18 (-1.50, 5.86) | **CD+ESWT** |  |  |  |  |  |  |
| **4.90 (0.21, 9.59)** | 2.72 (-3.24, 8.68) | **HBO+CD** |  |  |  |  |  |
| **3.79 (0.30, 7.28)** | 1.61 (-3.46, 6.68) | -1.11 (-4.24, 2.02) | **HBO** |  |  |  |  |
| 0.97 (-2.70, 4.65) | -1.21 (-5.62, 3.21) | -3.93 (-9.89, 2.03) | -2.82 (-7.89, 2.26) | **ESWT+HBO** |  |  |  |
| 1.26 (-0.69, 3.20) | -0.92 (-4.04, 2.20) | -3.64 (-8.72, 1.43) | -2.53 (-6.53, 1.47) | 0.28 (-2.84, 3.40) | **ESWT** |  |  |
| 0.76 (-1.21, 2.73) | -1.42 (-5.32, 2.47) | -4.14 (-9.23, 0.94) | -3.03 (-7.04, 0.98) | -0.22 (-4.11, 3.67) | -0.50 (-2.83, 1.82) | **CD+BMAC** |  |
| -1.34 (-3.46, 0.78) | -3.52 (-7.42, 0.37) | **-6.24 (-11.39, -1.10)** | **-5.13 (-9.22, -1.05)** | -2.32 (-6.21, 1.58) | **-2.60 (-4.93, -0.27)** | **-2.10 (-3.20, -1.00)** | **CD** |

**Note.** Cells report mean differences with 95% CI for the column intervention compared with the row intervention. Bold values indicate that the 95% CI excludes the null value (0). For VAS, lower values indicate greater pain relief.

**Supplementary Table S2. League table for Harris Hip Score (HHS).**

| **Placebo** |  |  |  |  |  |
| --- | --- | --- | --- | --- | --- |
| -5.83 (-19.65, 7.99) | **ESWT+HBO** |  |  |  |  |
| **-8.83 (-16.24, -1.42)** | -3.00 (-14.67, 8.66) | **ESWT** |  |  |  |
| -12.69 (-25.99, 0.61) | -6.86 (-22.92, 9.20) | -3.86 (-14.90, 7.18) | **CD+ESWT** |  |  |
| -2.36 (-10.94, 6.22) | 3.47 (-11.27, 18.21) | 6.47 (-2.54, 15.48) | 10.33 (-3.92, 24.58) | **CD+BMAC** |  |
| 5.92 (-3.00, 14.85) | 11.75 (-2.85, 26.36) | **14.76 (5.97, 23.54)** | **18.61 (4.50, 32.72)** | **8.28 (3.60, 12.97)** | **CD** |

**Note.** Cells report mean differences with 95% CI for the column intervention compared with the row intervention. Bold values indicate that the 95% CI excludes the null value (0). For HHS, higher values indicate better outcomes.

**Supplementary Table S3. League table for imaging progression evaluation.**

| **Placebo** |  |  |  |  |  |  |
| --- | --- | --- | --- | --- | --- | --- |
| 0.81 (0.07, 8.99) | **HBO+CD** |  |  |  |  |  |
| 2.26 (0.34, 15.07) | 2.80 (0.35, 22.49) | **HBO** |  |  |  |  |
| 7.16 (0.70, 73.76) | 8.89 (0.40, 196.52) | 3.17 (0.21, 47.98) | **ESWT+HBO** |  |  |  |
| **6.88 (1.52, 31.19)** | 8.53 (0.68, 107.82) | 3.05 (0.39, 23.79) | 0.96 (0.16, 5.67) | **ESWT** |  |  |
| **7.99 (1.91, 33.38)** | **9.92 (1.13, 87.10)** | 3.54 (0.73, 17.27) | 1.12 (0.10, 12.45) | 1.16 (0.23, 5.95) | **CD+BMAC** |  |
| 1.98 (0.56, 6.95) | 2.46 (0.31, 19.29) | 0.88 (0.21, 3.65) | 0.28 (0.03, 2.79) | 0.29 (0.07, 1.27) | **0.25 (0.12, 0.50)** | **CD** |

**Note.** Cells report odds ratios with 95% CI for the column intervention compared with the row intervention. Bold values indicate that the 95% CI excludes the null value (1). For imaging progression, odds ratios below 1 indicate lower risk of radiographic progression.

**Supplementary Table S4. League table for conversion to total hip arthroplasty (THA).**

| **Placebo** |  |  |  |  |
| --- | --- | --- | --- | --- |
| 12.26 (0.33, 455.14) | **ESWT+HBO** |  |  |  |
| 11.77 (0.76, 181.32) | 0.96 (0.09, 10.20) | **ESWT** |  |  |
| **3.57 (1.14, 11.21)** | 0.29 (0.01, 9.41) | 0.30 (0.02, 3.88) | **CD+BMAC** |  |
| 2.87 (0.83, 9.88) | 0.23 (0.01, 6.98) | 0.24 (0.02, 2.79) | 0.80 (0.39, 1.67) | **CD** |

**Note.** Cells report odds ratios with 95% CI for the column intervention compared with the row intervention. Bold values indicate that the 95% CI excludes the null value (1). For THA conversion, odds ratios below 1 indicate lower risk of conversion to total hip arthroplasty.

**Supplementary Table S5. League table for Oxford Hip Score (OHS).**

| **Placebo** |  |  |  |
| --- | --- | --- | --- |
| **-7.71 (-11.02, -4.39)** | **HBO+CD** |  |  |
| **-5.89 (-9.16, -2.61)** | 1.82 (-0.03, 3.68) | **HBO** |  |
| **-5.30 (-7.78, -2.82)** | **2.41 (0.21, 4.61)** | 0.59 (-1.55, 2.72) | **CD** |

**Note.** Cells report mean differences with 95% CI for the column intervention compared with the row intervention. Bold values indicate that the 95% CI excludes the null value (0). For OHS, higher values indicate better outcomes.

**Supplementary Table S6. League table for SF-36.**

| **CD** |  |  |
| --- | --- | --- |
| **-4.01 (-6.38, -1.63)** | **HBO+CD** |  |
| -2.10 (-4.79, 0.60) | 1.91 (-0.15, 3.96) | **HBO** |

**Note.** Cells report mean differences with 95% CI for the column intervention compared with the row intervention. Bold values indicate that the 95% CI excludes the null value (0). For SF-36, higher values indicate better outcomes.

**Supplementary Table S7. League table for adverse events.**

| **Placebo** |  |  |  |  |
| --- | --- | --- | --- | --- |
| 0.01 (0.00, 1.16) | **HBO** |  |  |  |
| **0.04 (0.00, 0.28)** | 3.44 (0.05, 234.18) | **ESWT** |  |  |
| **0.01 (0.00, 0.08)** | 0.58 (0.01, 34.80) | **0.17 (0.03, 0.83)** | **CD+BMAC** |  |
| **0.01 (0.00, 0.13)** | 1.09 (0.02, 59.40) | 0.32 (0.08, 1.21) | 1.87 (0.79, 4.40) | **CD** |

**Note.** Cells report odds ratios with 95% CI for the column intervention compared with the row intervention. Bold values indicate that the 95% CI excludes the null value (1). For adverse events, odds ratios below 1 indicate fewer adverse events.
