# Supplementary material for: Comparative efficacy and safety of core decompression, cell-based therapy, hyperbaric oxygen therapy, extracorporeal shock wave therapy, and combined regimens for osteonecrosis of the femoral head: a network meta-analysis
Source: Front Cell Dev Biol. 2026 Jul 15;14:1876711. doi: 10.3389/fcell.2026.1876711 (PMC13416348; doi:10.3389/fcell.2026.1876711)
Supplement: Supplementary file 5 [file Table4.docx]

**Supplementary Table S8-S14: SUCRA Ranking Results**

**General note.** The following SUCRA ranking tables are presented in the order specified for the manuscript: VAS, HHS, imaging progression evaluation, THA conversion, OHS, SF-36, and adverse events. SUCRA values and PrBest are reported as percentages. Higher SUCRA values indicate a greater probability that the intervention ranks among the most favorable options for the outcome direction specified in each table. MeanRank represents the average treatment rank; smaller values indicate more favorable average ranking. Ranking probabilities should be interpreted together with the corresponding effect estimates, confidence intervals, heterogeneity, inconsistency, and network geometry.

**Abbreviations:** BMAC, denotes the pooled analytical category for cell therapy; CD, core decompression; ESWT, extracorporeal shock wave therapy; HBO, hyperbaric oxygen therapy; HHS, Harris Hip Score; OHS, Oxford Hip Score; PrBest, probability of being the best-ranked treatment; SF-36, 36-item Short Form Health Survey; SUCRA, surface under the cumulative ranking curve; THA, total hip arthroplasty; VAS, visual analogue scale.

**Summary of top-ranked interventions by SUCRA**

| **Outcome** | **Top-ranked intervention** | **SUCRA (%)** | **PrBest (%)** |
| --- | --- | --- | --- |
| VAS | HBO+CD | 89.9 | 64.3 |
| HHS | CD+ESWT | 88.4 | 66.4 |
| Imaging progression evaluation | CD+BMAC | 83.6 | 39.1 |
| THA conversion | ESWT | 78.7 | 39.6 |
| OHS | HBO+CD | 98.6 | 96.1 |
| SF-36 | HBO+CD | 98.2 | 96.4 |
| Adverse events | Placebo | 99.3 | 97.3 |

**Supplementary Table S8. SUCRA ranking probabilities for visual analogue scale (VAS).**

| **Rank** | **Treatment** | **SUCRA (%)** | **PrBest (%)** | **MeanRank** |
| --- | --- | --- | --- | --- |
| **1** | **HBO+CD** | **89.9** | **64.3** | **1.7** |
| 2 | HBO | 80.6 | 16.7 | 2.4 |
| 3 | CD+ESWT | 64.2 | 13.2 | 3.5 |
| 4 | ESWT | 50.9 | 0.7 | 4.4 |
| 5 | ESWT+HBO | 44.1 | 4.3 | 4.9 |
| 6 | CD+BMAC | 42.0 | 0.8 | 5.1 |
| 7 | Placebo | 24.0 | 0.0 | 6.3 |
| 8 | CD | 4.3 | 0.0 | 7.7 |

**Note.** For VAS, lower values indicate greater pain relief; rankings were interpreted according to the favorable direction specified in the network meta-analysis. Values are ordered from the highest to the lowest SUCRA value. The first-ranked treatment is highlighted in green.

**Supplementary Table S9. SUCRA ranking probabilities for Harris Hip Score (HHS).**

| **Rank** | **Treatment** | **SUCRA (%)** | **PrBest (%)** | **MeanRank** |
| --- | --- | --- | --- | --- |
| **1** | **CD+ESWT** | **88.4** | **66.4** | **1.6** |
| 2 | ESWT | 77.0 | 16.0 | 2.1 |
| 3 | ESWT+HBO | 58.4 | 14.9 | 3.1 |
| 4 | CD+BMAC | 43.9 | 2.5 | 3.8 |
| 5 | Placebo | 28.8 | 0.1 | 4.6 |
| 6 | CD | 3.3 | 0.0 | 5.8 |

**Note.** For HHS, higher values indicate better hip function. Values are ordered from the highest to the lowest SUCRA value. The first-ranked treatment is highlighted in green.

**Supplementary Table S10. SUCRA ranking probabilities for imaging progression evaluation.**

| **Rank** | **Treatment** | **SUCRA (%)** | **PrBest (%)** | **MeanRank** |
| --- | --- | --- | --- | --- |
| **1** | **CD+BMAC** | **83.6** | **39.1** | **2.0** |
| 2 | ESWT | 78.0 | 20.4 | 2.3 |
| 3 | ESWT+HBO | 75.1 | 37.3 | 2.5 |
| 4 | HBO | 43.6 | 2.4 | 4.4 |
| 5 | CD | 37.9 | 0.0 | 4.7 |
| 6 | Placebo | 16.3 | 0.0 | 6.0 |
| 7 | HBO+CD | 15.5 | 0.8 | 6.1 |

**Note.** For imaging progression, lower risk of radiographic deterioration indicates a more favorable outcome. Values are ordered from the highest to the lowest SUCRA value. The first-ranked treatment is highlighted in green.

**Supplementary Table S11. SUCRA ranking probabilities for conversion to total hip arthroplasty (THA).**

| **Rank** | **Treatment** | **SUCRA (%)** | **PrBest (%)** | **MeanRank** |
| --- | --- | --- | --- | --- |
| **1** | **ESWT** | **78.7** | **39.6** | **1.9** |
| 2 | ESWT+HBO | 74.4 | 47.3 | 2.0 |
| 3 | CD+BMAC | 53.1 | 10.5 | 2.9 |
| 4 | CD | 39.2 | 2.4 | 3.4 |
| 5 | Placebo | 4.6 | 0.1 | 4.8 |

**Note.** For THA conversion, lower risk of conversion to total hip arthroplasty indicates a more favorable outcome. Values are ordered from the highest to the lowest SUCRA value. The first-ranked treatment is highlighted in green.

**Supplementary Table S12. SUCRA ranking probabilities for Oxford Hip Score (OHS).**

| **Rank** | **Treatment** | **SUCRA (%)** | **PrBest (%)** | **MeanRank** |
| --- | --- | --- | --- | --- |
| **1** | **HBO+CD** | **98.6** | **96.1** | **1.0** |
| 2 | HBO | 57.9 | 2.5 | 2.3 |
| 3 | CD | 43.5 | 1.4 | 2.7 |
| 4 | Placebo | 0.0 | 0.0 | 4.0 |

**Note.** For OHS, higher values indicate better hip-related function. Values are ordered from the highest to the lowest SUCRA value. The first-ranked treatment is highlighted in green.

**Supplementary Table S13. SUCRA ranking probabilities for SF-36.**

| **Rank** | **Treatment** | **SUCRA (%)** | **PrBest (%)** | **MeanRank** |
| --- | --- | --- | --- | --- |
| **1** | **HBO+CD** | **98.2** | **96.4** | **1.0** |
| 2 | HBO | 48.6 | 3.6 | 2.0 |
| 3 | CD | 3.2 | 0.0 | 2.9 |

**Note.** For SF-36, higher values indicate better health-related quality of life. Values are ordered from the highest to the lowest SUCRA value. The first-ranked treatment is highlighted in green.

**Supplementary Table S14. SUCRA ranking probabilities for adverse events.**

| **Rank** | **Treatment** | **SUCRA (%)** | **PrBest (%)** | **MeanRank** |
| --- | --- | --- | --- | --- |
| **1** | **Placebo** | **99.3** | **97.3** | **1.0** |
| 2 | ESWT | 66.6 | 0.0 | 2.3 |
| 3 | CD | 37.2 | 0.0 | 3.5 |
| 4 | HBO | 34.6 | 2.6 | 3.6 |
| 5 | CD+BMAC | 12.3 | 0.0 | 4.5 |

**Note.** For adverse events, lower odds of adverse events indicate a more favorable safety profile. Values are ordered from the highest to the lowest SUCRA value. The first-ranked treatment is highlighted in green.
