# Supplementary material for: Comparative efficacy and safety of core decompression, cell-based therapy, hyperbaric oxygen therapy, extracorporeal shock wave therapy, and combined regimens for osteonecrosis of the femoral head: a network meta-analysis
Source: Front Cell Dev Biol. 2026 Jul 15;14:1876711. doi: 10.3389/fcell.2026.1876711 (PMC13416348; doi:10.3389/fcell.2026.1876711)
Supplement: Supplementary file 6 [file DataSheet1.docx]

**Supplementary Figures S1–S11**

**=**
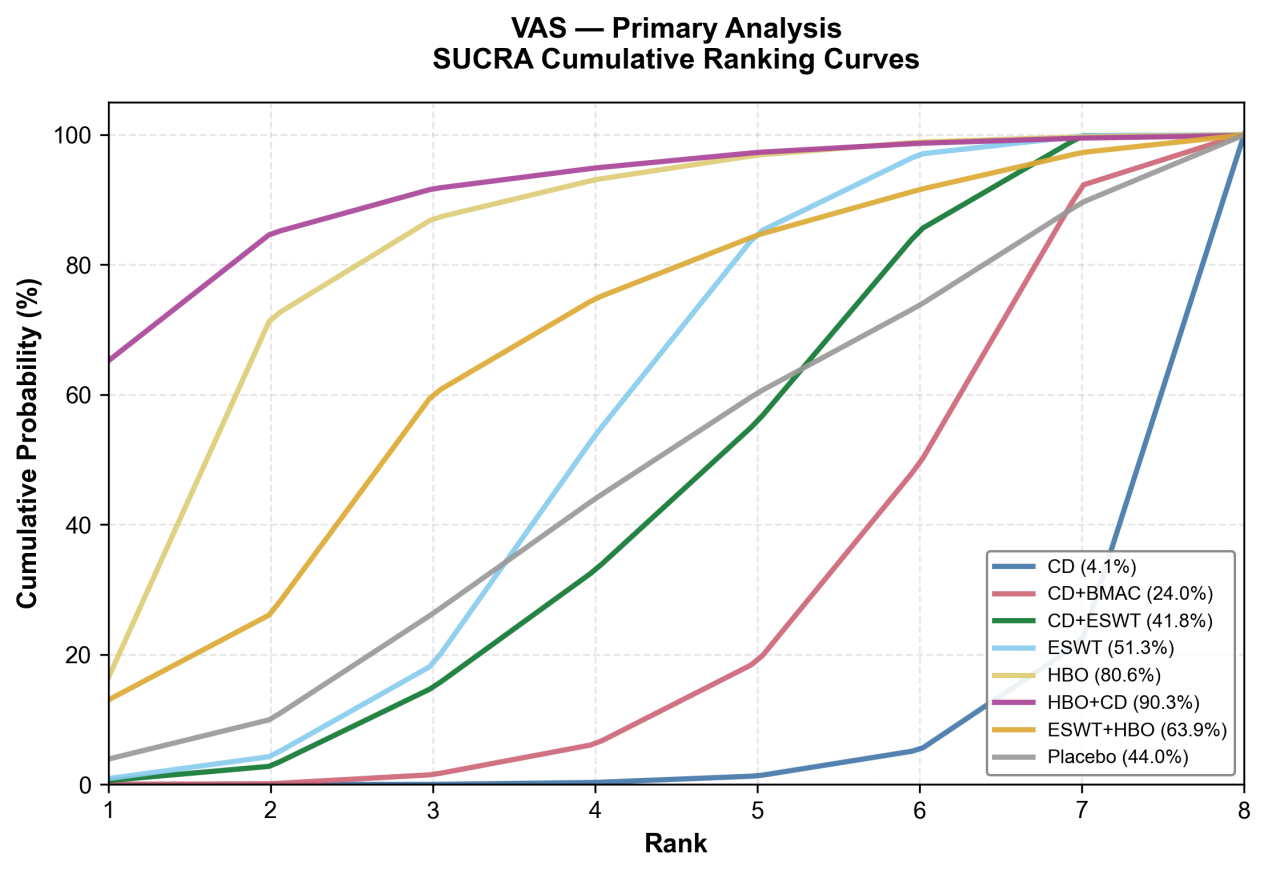


**FigS1.**VAS Primary curves


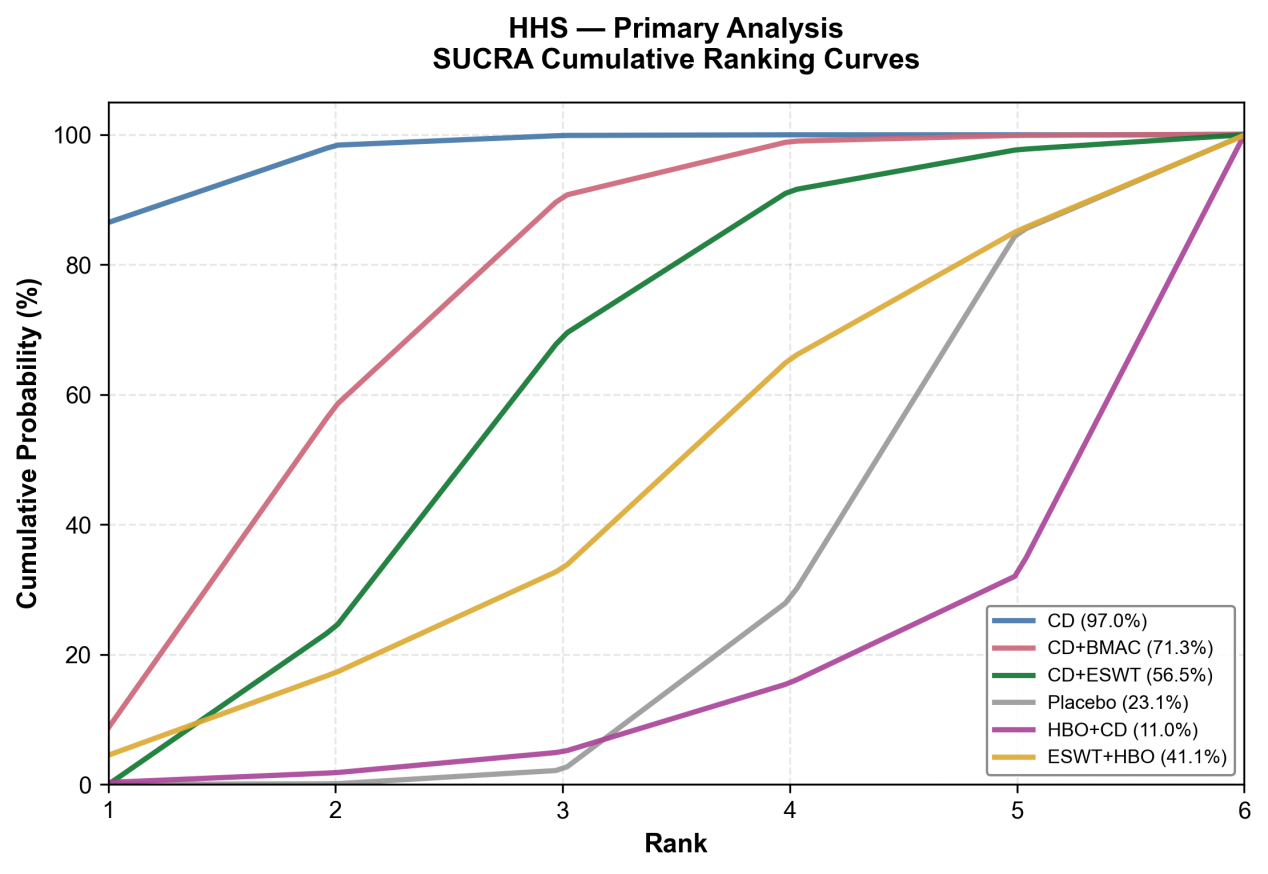


**FigS2.**HHS Primary curves


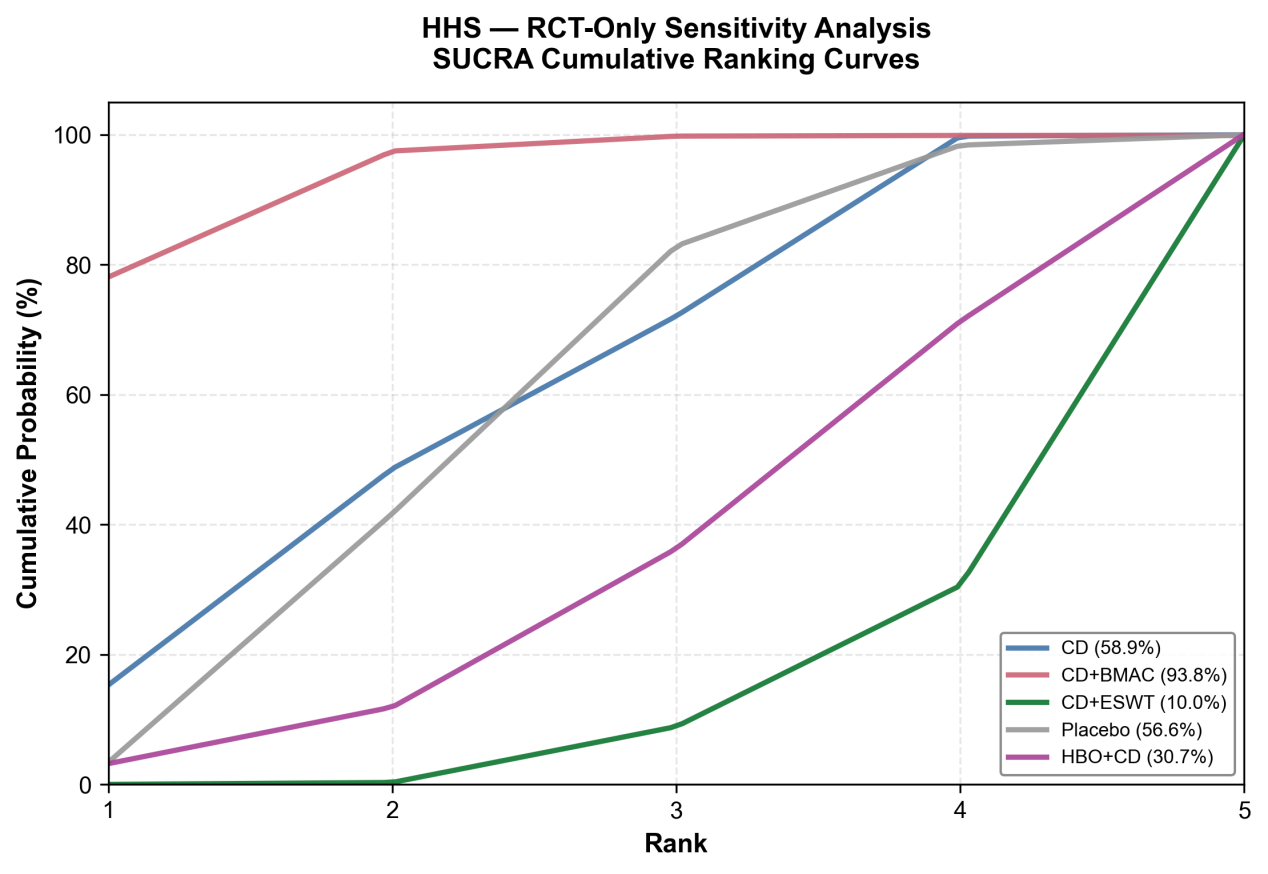


**FigS3.**HHS RCT curves


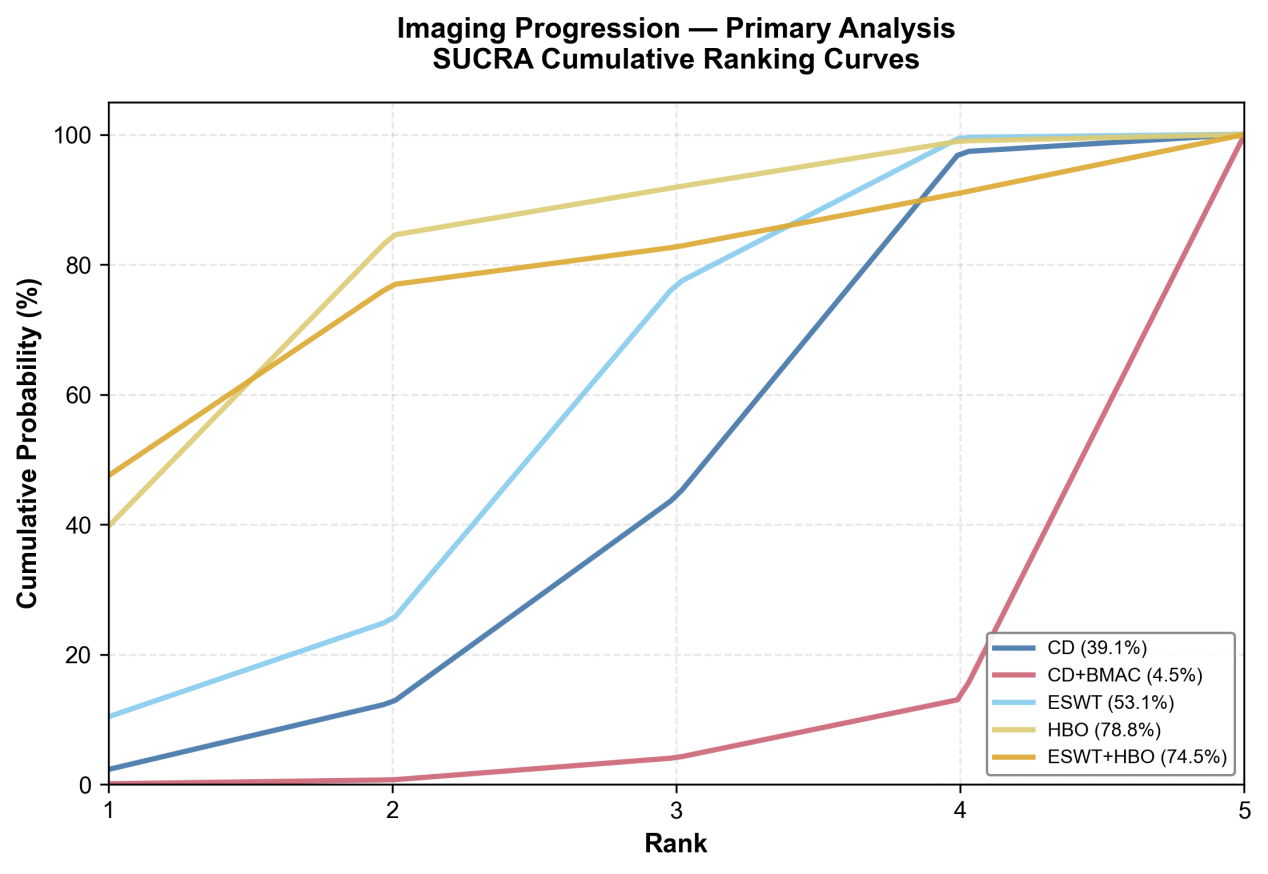


**FigS4.**Imaging Primary curves


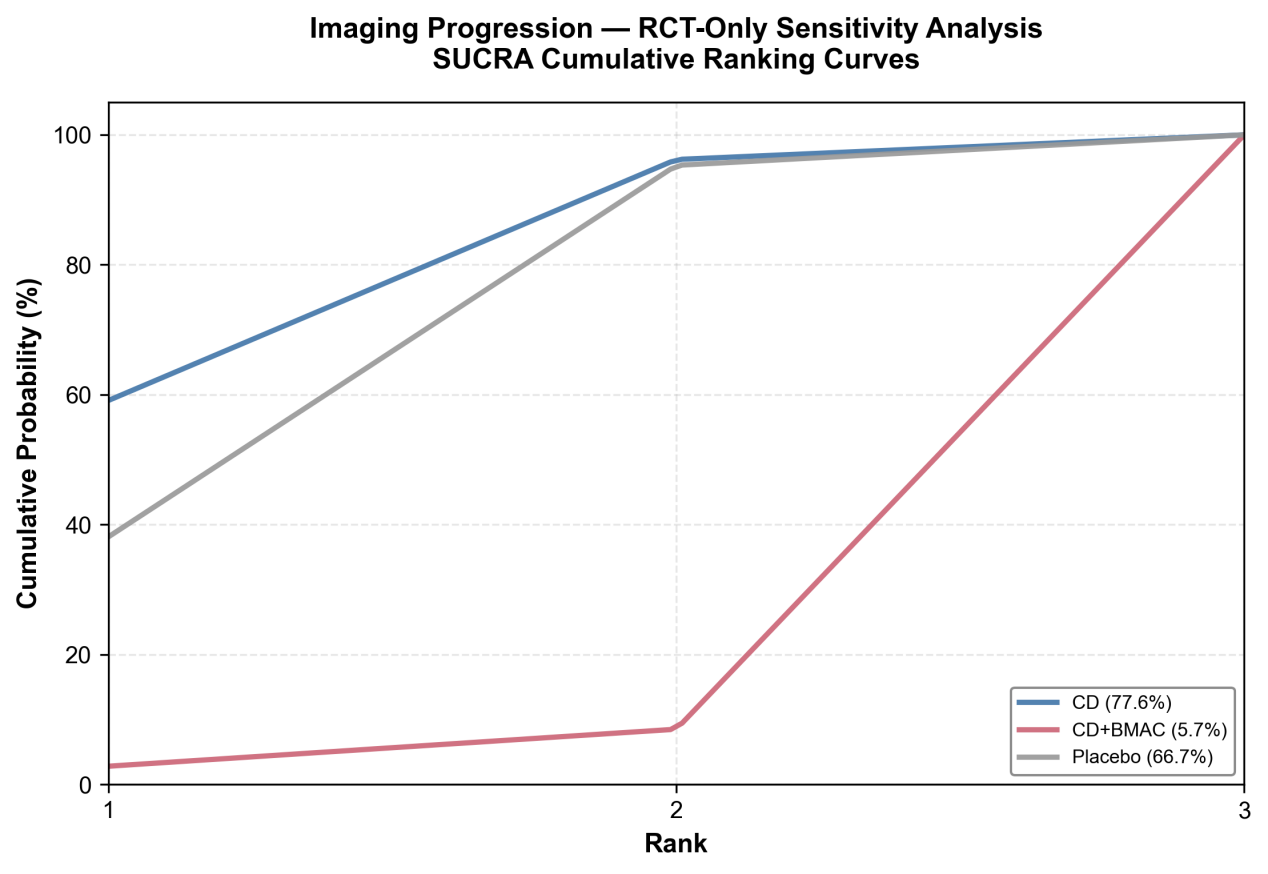


**FigS5.**Imaging RCT curves


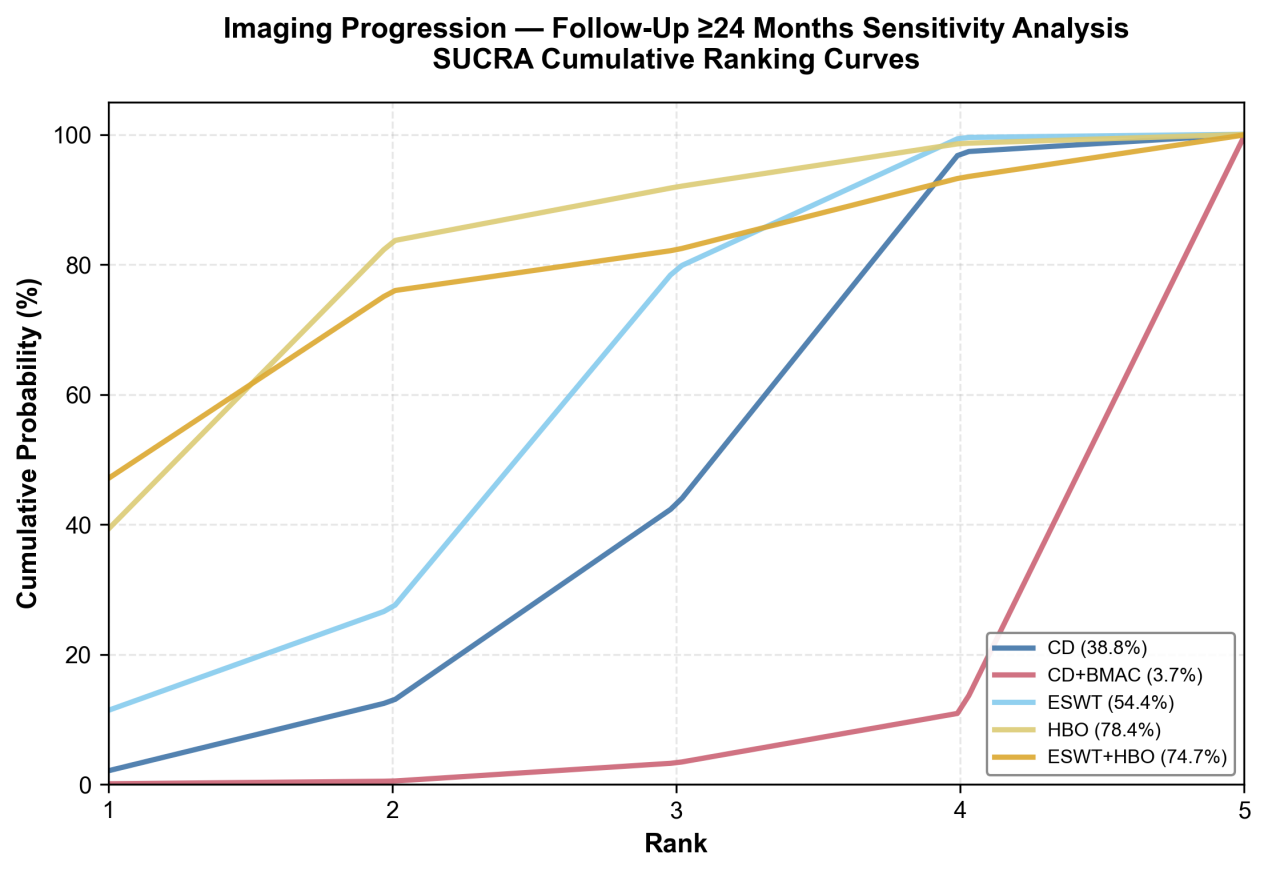


**FigS6.**Imaging FU24 curves


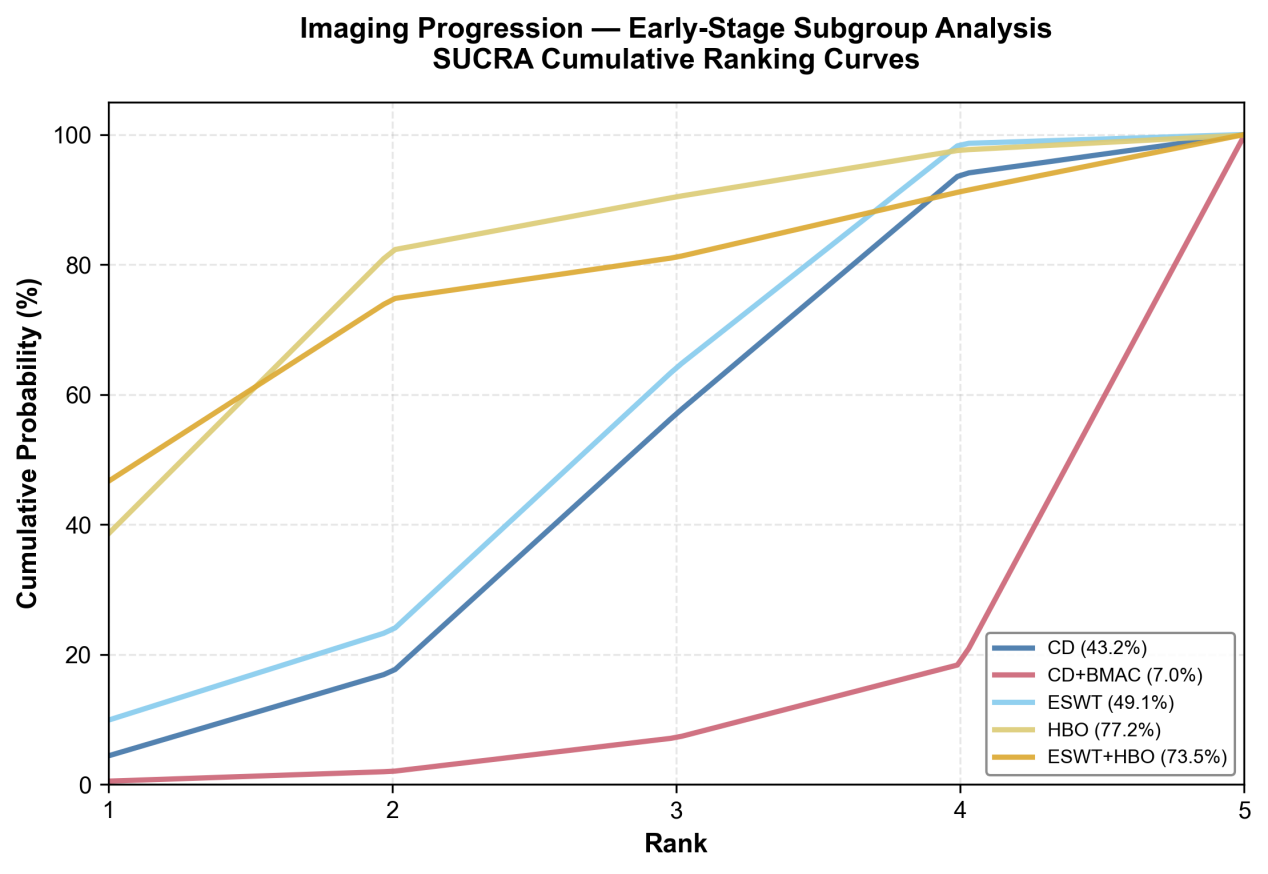


**FigS7.**Imaging Early curves


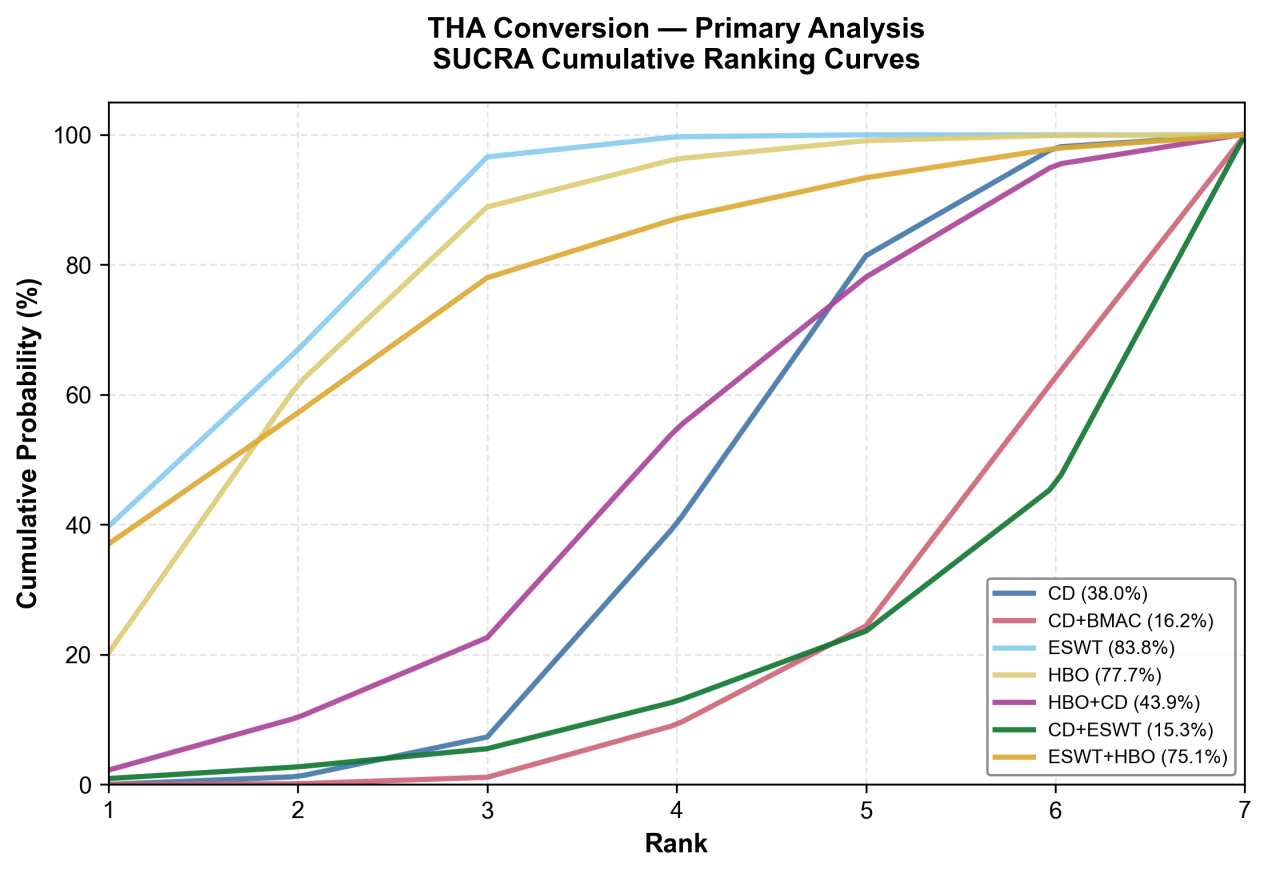


**FigS8.**THA Primary curves


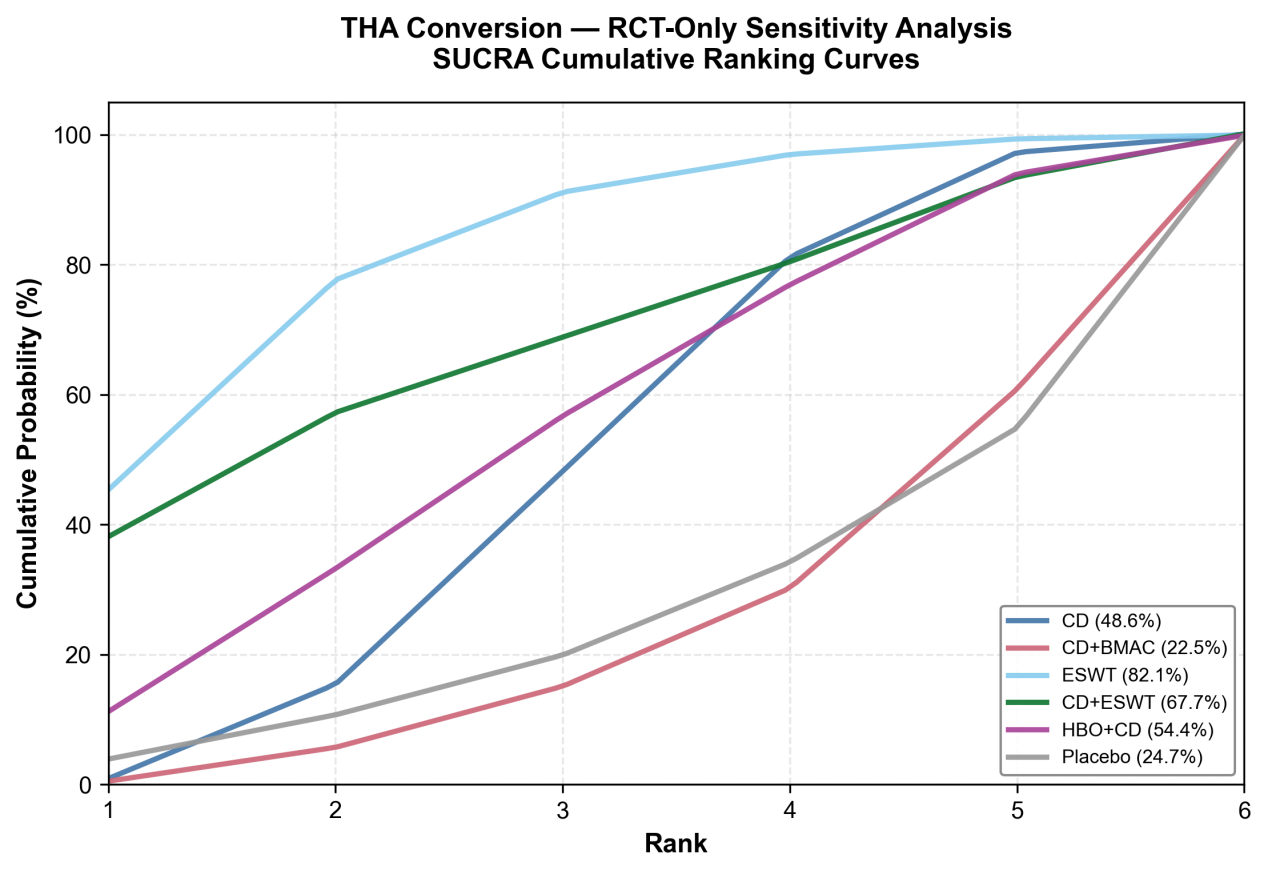


**FigS9.**THA RCT curves


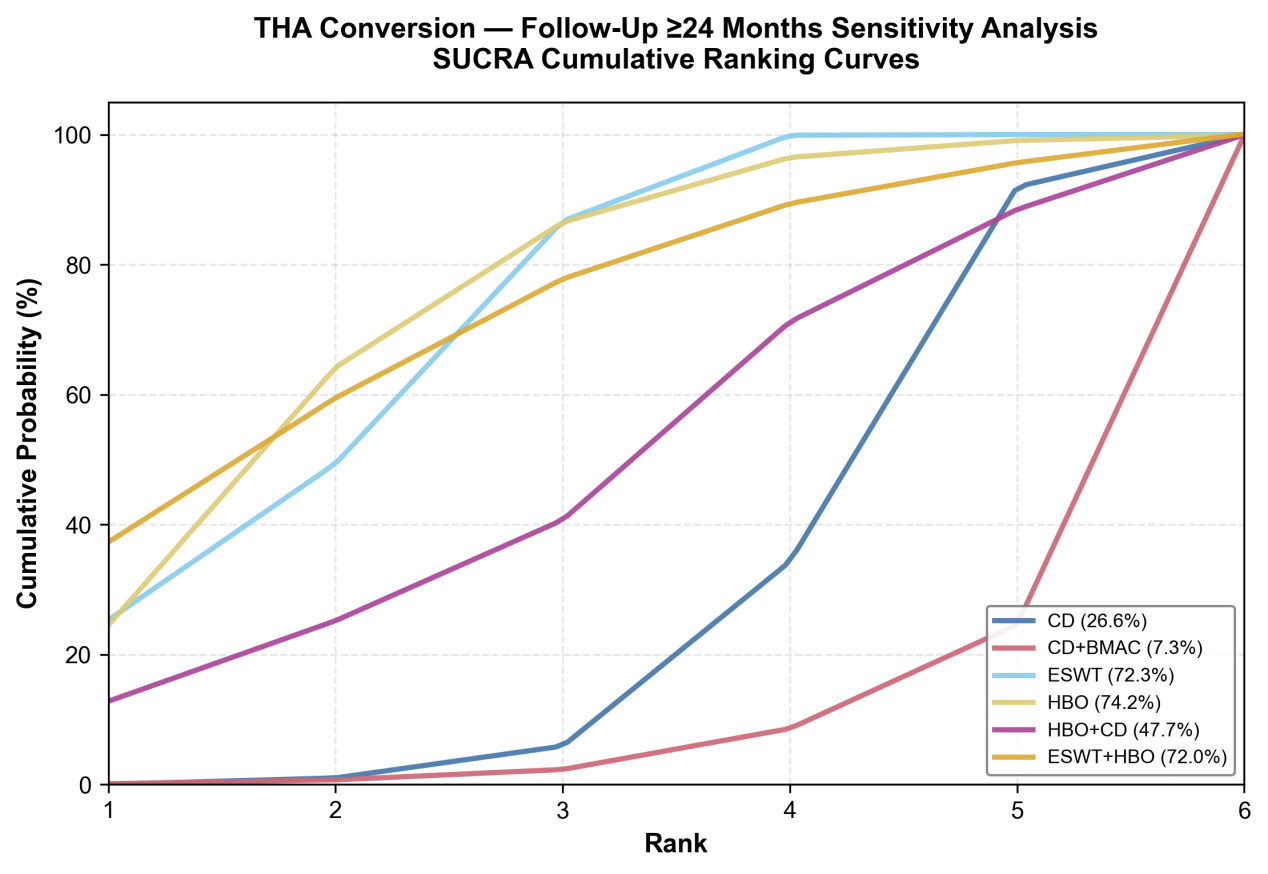


**FigS10.**THA FU24 curves


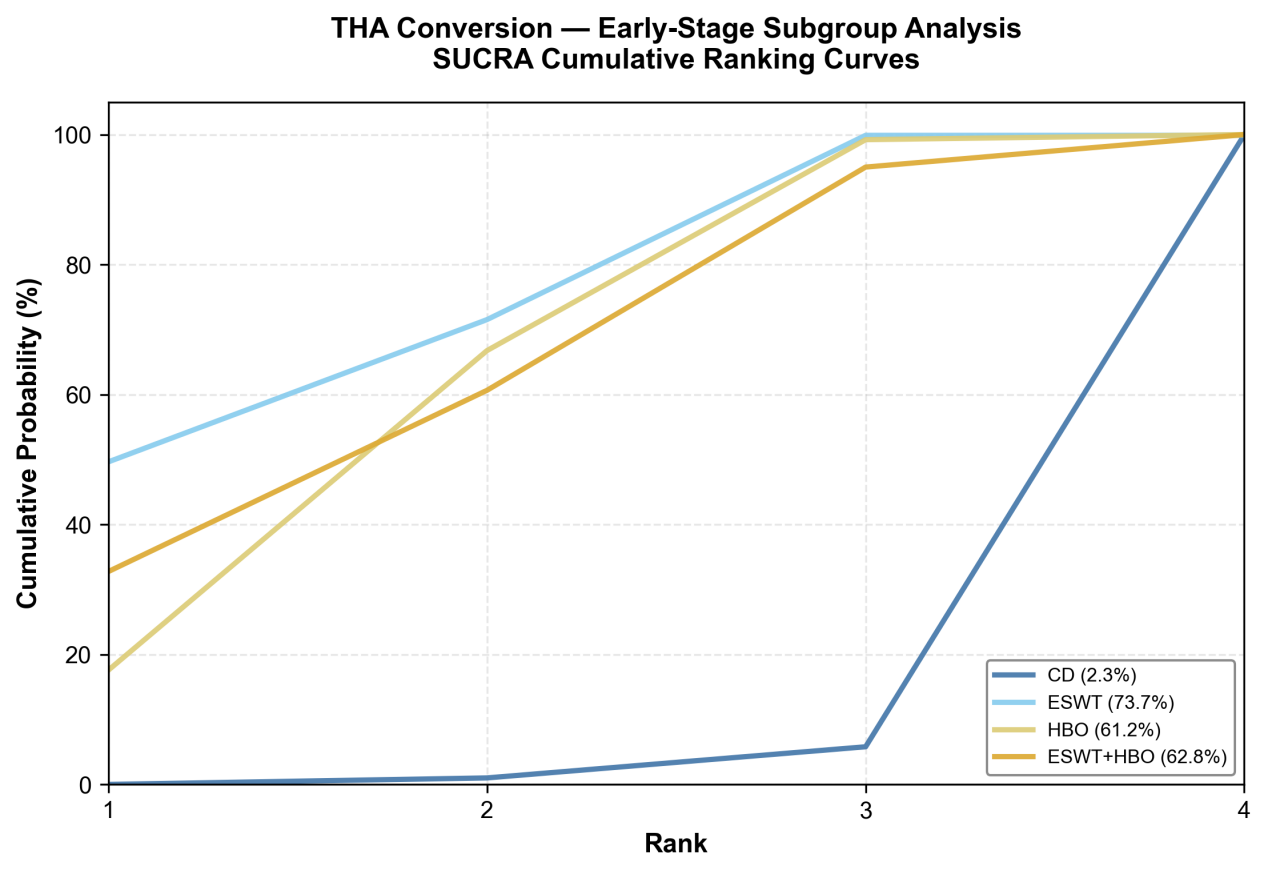


**FigS11.**THA Early curves
